# Engineering of cytosine base editors with DNA damage minimization and editing scope diversification

**DOI:** 10.1093/nar/gkad855

**Published:** 2023-10-16

**Authors:** Bo Yuan, Shuqian Zhang, Liting Song, Jinlong Chen, Jixin Cao, Jiayi Qiu, Zilong Qiu, Jingqi Chen, Xing-Ming Zhao, Tian-Lin Cheng

**Affiliations:** Institute of Pediatrics, National Children's Medical Center, Children's Hospital, Institute for Translational Brain Research, State Key Laboratory of Medical Neurobiology, MOE Frontiers Center for Brain Science, Fudan University, Shanghai 200032, China; Institute of Neuroscience, State Key Laboratory of Neuroscience, CAS Center for excellence in Brain Science and Intelligence Technology, Chinese Academy of Sciences, Shanghai 200031, China; Institute of Pediatrics, National Children's Medical Center, Children's Hospital, Institute for Translational Brain Research, State Key Laboratory of Medical Neurobiology, MOE Frontiers Center for Brain Science, Fudan University, Shanghai 200032, China; Department of Pediatrics, Qilu Hospital of Shandong University, Ji’nan 250012, China; Institute of Science and Technology for Brain-inspired Intelligence, Key Laboratory of Computational Neuroscience and Brain-Inspired Intelligence, Fudan University, Shanghai 200433, China; National Clinical Research Center for Aging and Medicine, Huashan Hospital, Fudan University, Shanghai 200040, China; Institute of Pediatrics, National Children's Medical Center, Children's Hospital, Institute for Translational Brain Research, State Key Laboratory of Medical Neurobiology, MOE Frontiers Center for Brain Science, Fudan University, Shanghai 200032, China; Institute of Science and Technology for Brain-inspired Intelligence, Key Laboratory of Computational Neuroscience and Brain-Inspired Intelligence, Fudan University, Shanghai 200433, China; Institute of Pediatrics, National Children's Medical Center, Children's Hospital, Institute for Translational Brain Research, State Key Laboratory of Medical Neurobiology, MOE Frontiers Center for Brain Science, Fudan University, Shanghai 200032, China; Institute of Neuroscience, State Key Laboratory of Neuroscience, CAS Center for excellence in Brain Science and Intelligence Technology, Chinese Academy of Sciences, Shanghai 200031, China; National Clinical Research Center for Aging and Medicine, Huashan Hospital, Fudan University, Shanghai 200040, China; Songjiang Hospital, Songjiang Institute, Shanghai Jiao Tong University School of Medicine, Shanghai, China; Institute of Science and Technology for Brain-inspired Intelligence, Key Laboratory of Computational Neuroscience and Brain-Inspired Intelligence, Fudan University, Shanghai 200433, China; State Key Laboratory of Medical Neurobiology, Institutes of Brain Science, Fudan University, Shanghai, China; MOE Key Laboratory of Computational Neuroscience and Brain-Inspired Intelligence, and MOE Frontiers Center for Brain Science, Fudan University, Shanghai, China; Institute of Science and Technology for Brain-inspired Intelligence, Key Laboratory of Computational Neuroscience and Brain-Inspired Intelligence, Fudan University, Shanghai 200433, China; State Key Laboratory of Medical Neurobiology, Institutes of Brain Science, Fudan University, Shanghai, China; MOE Key Laboratory of Computational Neuroscience and Brain-Inspired Intelligence, and MOE Frontiers Center for Brain Science, Fudan University, Shanghai, China; Institute of Pediatrics, National Children's Medical Center, Children's Hospital, Institute for Translational Brain Research, State Key Laboratory of Medical Neurobiology, MOE Frontiers Center for Brain Science, Fudan University, Shanghai 200032, China

## Abstract

Cytosine base editors (CBEs), which enable precise C-to-T substitutions, have been restricted by potential safety risks, including DNA off-target edits, RNA off-target edits and additional genotoxicity such as DNA damages induced by double-strand breaks (DSBs). Though DNA and RNA off-target edits have been ameliorated via various strategies, evaluation and minimization of DSB-associated DNA damage risks for most CBEs remain to be resolved. Here we demonstrate that YE1, an engineered CBE variant with minimized DNA and RNA off-target edits, could induce prominent DSB-associated DNA damage risks, manifested as γH2AX accumulation in human cells. We then perform deaminase engineering for two deaminases lamprey LjCDA1 and human APOBEC3A, and generate divergent CBE variants with eliminated DSB-associated DNA damage risks, in addition to minimized DNA/RNA off-target edits. Furthermore, the editing scopes and sequence preferences of APOBEC3A-derived CBEs could be further diversified by internal fusion strategy. Taken together, this study provides updated evaluation platform for DSB-associated DNA damage risks of CBEs and further generates a series of safer toolkits with diversified editing signatures to expand their applications.

## Graphical Abstract

**Graphical Abstract:**
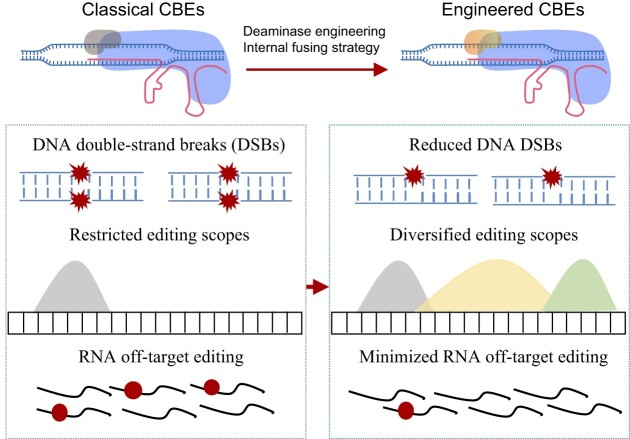

## Introduction

Cytosine base editors (CBEs), which are generally composed of cytidine deaminases and nicking or deactivated Cas nucleases (nCas9 or dCas9) (1–4,) could mediate precise and efficient C-to-T substitutions in targeted genomic DNA regions. Safety risks are critical challenges that need to be addressed to expand their applications (5,6). Generally, DNA and RNA off-target edits are considered as the major safety challenges of CBEs (5–7), which are mainly associated with the core biochemical properties of Cas and deaminases. For instance, high mismatch tolerance of Cas proteins would lead to reduced binding specificity and Cas-dependent DNA off-target edits (8), while cytosine deamination activity of deaminases at DNA or RNA level might induce DNA or RNA off-target edits (5–7). To reduce off-target editing risks, CBEs have been optimized via various strategies in recent years. For example, cytidine deaminases of the AID/APOBEC family, including APOBEC1, APOBEC3A, APOBEC3G and lamprey CDAs, have been exploited and mutated to generate functional CBEs with minimized DNA and RNA off-target edits (5,6,9–14). Additionally, internal fusion strategies have also been explored to reduce DNA and RNA off-target edits of base editors (15–17).

RNA off-target editing analysis of base editors was commonly evaluated by GATK HaplotypeCaller tool. However, it has been demonstrated that HaplotypeCaller tool lacked sensitivity to detect A-to-I RNA edits with <10% efficiency, while MuTect2 was a more sensitive tool for such analysis (18). Therefore, it is possible that RNA off-target edits of CBEs might be underestimated previously and re-analysis with MuTect2 is necessary.

It is also known that cytidine deaminases could induce DNA damages by triggering double-strand breaks (DSBs) (19–21), which could further stimulate undesired DNA damage response (DDR). Consistently, it has been reported that several CBEs induced obvious DSB-associated DNA damage risks, which was mainly attributed to deaminase domain (22,23). However, DSB-associated DNA damage risks of CBEs were usually compared with Cas9 in previous studies (23), which might lead to underestimation in consideration of robust DSB-associated DNA damage risks induced by Cas9 (24,25). As CBEs were composed of cytidine deaminases and nCas9, comparison between CBEs and nCas9 could provide more accurate quantification for deaminase-induced DSB-associated DNA damage risks, which would be valuable to guide subsequent DNA damage minimization. Recent work also reported an updated platform to evaluate DSB-associated DNA damage risks induced by CBEs by flow cytometry to quantify γH2AX accumulation, marker of DSBs and DNA damage risks, with deaminase-inactive CBEs as negative control (22), providing valuable insights into the evaluation and minimization of DSB-associated DNA damage risks.

Here, we demonstrated that an engineered CBE variant YE1 (10,11,26), which was considered as an optimized version with minimized DNA and RNA off-target edits, could induce prominent DSB-associated DNA damage risks in human cells. To develop improved CBE variants with both minimized DNA/RNA off-target edits and reduced DSB-associated DNA damage risks, we performed deaminase engineering for two cytidine deaminases lamprey LjCDA1 and human APOBEC3A, and finally generated engineered CBE variants with multidimensional safety improvement including DNA/RNA off-target editing minimization and DSB-associated DNA damage amelioration. Additionally, the editing scopes and sequence preferences of engineered CBEs in this study were further diversified without sacrificing safety through internal fusion strategy, which would be important for the widespread applications of CBEs in the future.

## Materials and methods

### Plasmid construction

Universal expression vector of base editors was derived from PX461 (Addgene #48140), in which the U6-sgRNA cassette was deleted. Then deaminase insertion sites (containing SpeI-BamHI-XbaI multiple cloning sequences) were created at N-/C- or internal regions of nCas9 via mutagenesis strategy (KOD-plus, Toyobo). Variant deaminase templates were amplified using high-fidelity DNA polymerase (KOD-plus, Toyobo) and inserted into expression vector by restriction cloning. Templates for rAPOBEC1 and APOBEC3A were derived from pCMV-BE3 (Addgene #73021) and HEK293T cDNAs respectively. Templates for lamprey cytidine deaminases have been described previously (13). Mutagenesis strategy (KOD-plus, Toyobo) was used to induce specific mutations for corresponding base editors. Plasmids expressing dSaCas9-UGI-T2A-mCherry and U6-sgsaRNA, which were used in R-loop assay, were derived from PX602 (Addgene #61593), in which D10A and N580A were induced via mutagenesis strategy (KOD-plus, Toyobo), and then UGI-T2A-mCherry cassette was inserted through BamHI-EcoRI double digestion strategy. Plasmids expressing sgRNAs were generated using U6-sgRNA-EF1alpha-UGI-T2A-mCherry as described previously. Additionally, mCherry was replaced with puromycin resistance gene to generate plasmid expressing sgRNAs for puromycin-based enrichment. Information for sgRNAs used in this study was listed in Supplementary Table S1.

### Human cell culture and transient transfection

HEK293T cells (Cell Bank of the Chinese Academy of Sciences, Shanghai, China) were cultured in Dulbecco's modified Eagle's medium (DMEM, Sigma-Aldrich) supplemented with 10% FBS (Gibco, Thermo Fisher Scientific) at 37°C in 5% CO_2_ incubator (Heraeus, Thermo Fisher Scientific). HEK293T cells were passaged and plated into 6-well or 48-well plates (Corning) for transfection. To characterize potential transcriptomic changes induced by DNA base editors, plasmid expressing base editor (with 2A-GFP), was co-transfected with plasmid expressing scrambled single guide RNA (sgRNA) and uracil glycosylase inhibitor (UGI) (with 2A-puromycin-resistant gene) into HEK293T cells. Base editor- and sgRNA-expressing plasmid (mole ratio 2:1) were mixed with 2.5 μl (48-well) or 8 μl (6-well) Lipo293^TM^ (Beyotime biotechnology, Shanghai, China) for transient transfection. For base editing analysis, 72 h after transfection, cells in 48-well plates were digested and prepared for flow cytometry to collect double positive cells. For transcriptome analysis, 24 h after transfection, medium was replaced with fresh DMEM containing 2 μg/ml puromycin (Beyotime biotechnology, Shanghai, China) for cells in 6-well plates. After culturing for another 48 h, cells were collected for RNA extraction and RNA sequencing.

### Base editing analysis

GFP/mcherry double-positive cells collected with flow cytrometry (Moflo XDP, Beckman Coulter/BD FACSAria™ Fusion Flow Cytometers) were treated with DirectPCR reagent (Viagene Biotech, Ningbo, China). Targeted amplifications were performed using LA Taq (Takara, Dalian, China), and base editing status was quantified with EditR software after Sanger sequencing (BGI, Shenzhen, China) or with CRISPResso2 after targeted amplicon sequencing (Shanghai Personalbio Technology, Shanghai, China). Targeted amplicon sequencing was performed using Illumina NovaSeq platform at Shanghai Personalbio Technology. Experiments for amplicon sequencing were performed in biological triplicates.

### Sequence preference analysis

C sites were initially classified according to their positions, and positions containing at least one C site with an average of >20% C-to-T editing frequency were included for sequence preference analysis. Then the impact of base types upstream or downstream of edited cytosine (NC or CN) was analyzed accordingly. For statistical analysis, one-tailed unpaired t-test was performed for comparison between every two groups and *P* < 0.05 was considered significant.

### GUIDE-seq analysis

To analyze the DSB number induced by different enzymes, we initially transfected nCas9, Cas9, N-YE1-BE or N-eA3A-RL1-BE with double-stranded oligodeoxynucleotide (dsODNs) into HEK293T cells by Nucleofector (Lonza). Cells were collected 3 days after transfection for genome isolation and GUIDE-seq analysis as described previously (27). For on-target DSB-induction analysis, nCas9, Cas9, N-YE1-BE or N-eA3A-RL1-BE with double-stranded oligodeoxynucleotide (dsODNs) and sgRNA-1 were transfected into HEK293T cells by Nucleofector. Cells were collected 3 days after transfection for genome isolation and targeted amplification with designed primers. The sequence of dsODNs was as follows: dsODN for: 5′- /5Phos/GTTTAATTGAGTTGTCATATGTTAATAACGGT*A*T-3′; dsODN rev: 5′-/5Phos/ATACCGTTATTAACATATGACAACTCAATTAA*A*C-3′.

### Single clone selection and whole genome sequencing

HEK293T cells were transfected with base editor- and sgRNA-expressing plasmids as described above. Here, sgRNA-expressing plasmid containing RNF2-targeting sgRNA and puro puromycin resistance gene. Medium was replaced with fresh DMEM containing 2 μg/ml puromycin (Beyotime biotechnology, Shanghai, China) for cells in 6-well plates every day for 4 days, and single cell clone was picked up into 96-well plate for further expansion. After culturing for 3–4 weeks, cellular genome DNA was purified using TGuide Cell/Tissue Genomic DNA Kit (TIANGEN Biotech) for whole genome sequencing using MGIseq platform (Genewiz).

### WGS data analysis

Paired-end sequencing reads were aligned to human genome build 38 (GRCh38/hg38) using BWA-MEM (v.0.7.17) (28). Aligned bams were sorted using GATK (v.4.2.4) tools MarkIlluminaAdapters, SamToFastq, MergeBamAlignment, SortSam. Optical duplicates were marked using GATK tools MarkDuplicates, SortSam and SetNmMdAndUqTags. Base quality recalibration was performed using GATK tools BaseRecalibrator and ApplyBQSR. All subsequent analyses were performed using high-performance computing cluster. Variant calling was conducted on every sample independently using Google DeepVariant with default parameter (29). HEK293T cells without any treatment were used as background, and identified SNVs were filtered from other samples. Variant statistics were performed using custom shell scripts and Excel.

### Genotoxicity analysis with γ-H2AX staining and flow cytometry

HEK293T cells were seeded into 48-well plates and transfected 24 h later. After culturing for another 72 h, cells were collected and washed with PBS, and fixed with 4% paraformaldehyde at room temperature (25 °C) for 15 min. Following washes with PBS, cells were permeabilized with 0.5% Triton X-100 in PBS for 2 min. Next, block cells with buffer (10% goat serum in TBS) at room temperature (25 °C) for 1 h. For the detection of DNA damage, stain the cells with γ-H2AX antibody (BD Pharmigen, 647) at 4°C for 2 h and wash with PBS. Cells were gated on fluorescein isothiocyanate and allophycocyanin using the Fortessa Flow Cytometer (BD Biosciences), and results were analyzed by FlowJo.

### R-loop assay for Cas9-independent DNA off-target analysis

R-loop assay was used to assess the Cas9-independent DNA off-target edits. In brief, Base editor-expressing plasmid was co-transfected with particular dSaCas9-UGI-T2A-mcherry-U6-sgsaRNA and cultured for 72 h. Double positive cells were collected by flow cytometry for targeted amplicon sequencing, and high-throughput sequencing data was analyzed using CRISPResso2.

### Transcriptome profiling for gene expression analysis and RNA editing quantification

Total RNA was isolated using Trizol reagent (Life technologies, Thermo Fisher Scientific) and RNA sequencing was performed using Illumina Novaseq (PE 2 ◊150), at a depth of ∼20 million reads per sample. RNA-seq data was analyzed following the long-RNA-seq-pipeline of ENCODE Consortium, and reads were aligned to GRCh38 reference genome using annotation GRCh38.v96 via STAR (v2.4.2a) in 2-pass mode (30). Sentieon® genomics tools (v202010.02) were used for variant calling. After removing duplicates with Picard (v2.23.6, http://broadinstitute.github.io/picard), reads were split at junctions into exon segments and reassigned the mapping qualities from STAR. To remove potential experimental biases, base quality score recalibration (BQSR) was performed as the DNA-seq. HaplotypeCaller tool from GATK (v4.1.4) (31) and MuTect2 was then used to identify variants, and VariantFiltration tool of GATK with parameter –filter-expression ‘QUAL < 25 || MQ < 20.0 || QD < 2.0 || FS > 30.0 || DP < 20′ was used to filter variants with base-quality score <25, mapping quality score <20, Fisher strand values >30.0, qual by depth values <2.0 or sequencing depth < 20. Variants found in at least two replicates of GFP-only expressing cells were considered as SNPs and filtered out from other base-editor-transfected groups. Statistical analysis was performed using two-tailed unpaired *t*-test and *P* < 0.05 was considered significant. Three to four independent biological replicates of each specific base editor were performed.

## Results

### Off-target edits and DNA damage risks induced by available CBE variants

**Figure 1.**
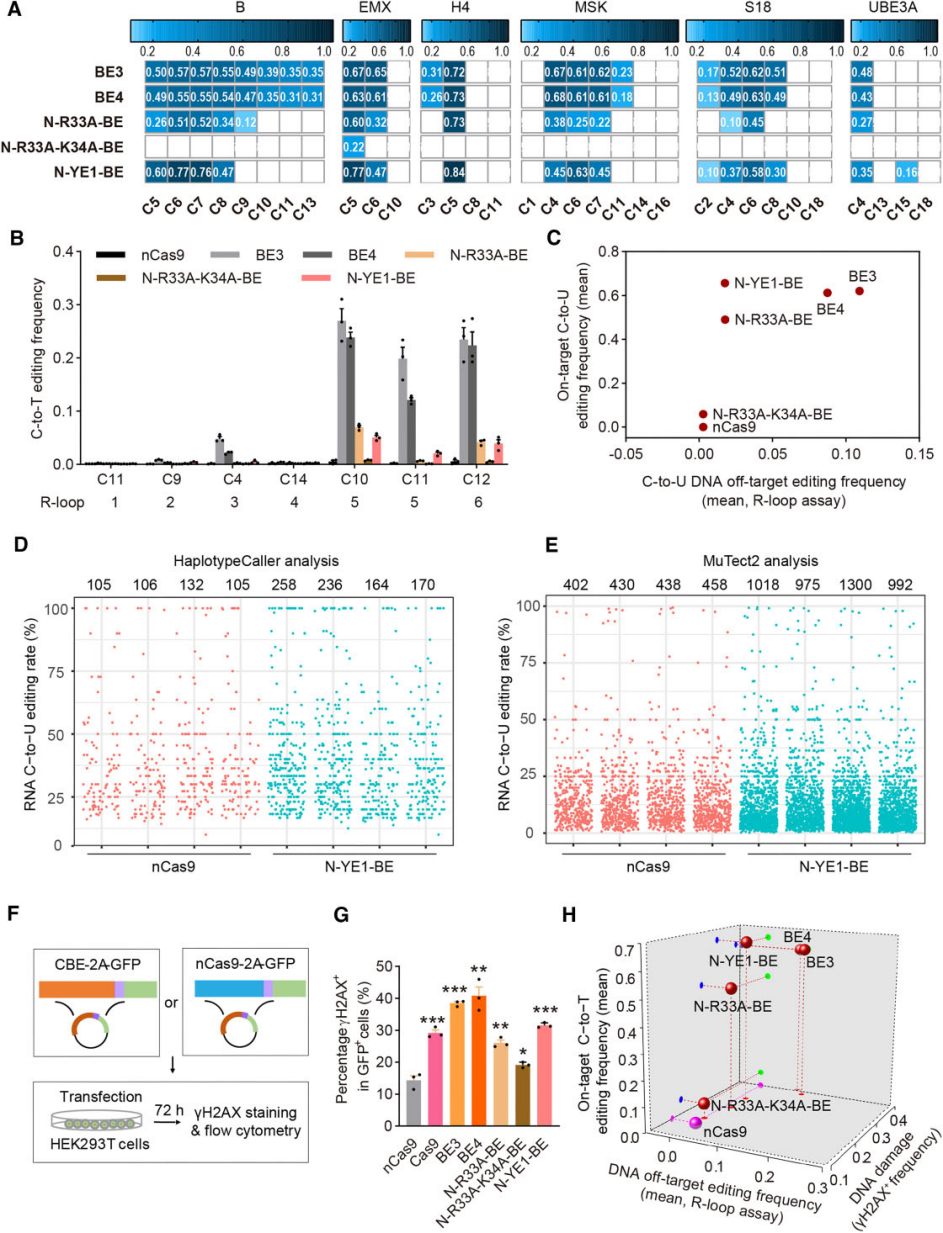
Editing signature, DNA/RNA off-target edits and DSB-associated DNA damage risks induced by N-YE1-BE. (**A**) Heatmaps showing on-target base editing efficiencies of BE3, BE4, N-R33A- BE, N-R33A-K34A-BE and N-YE1-BE across 6 sgRNAs (detailed sequences in Supplementary Table S1) in HEK293T cells (positive cells were collected by flow cytometry) in blue gradient color. *n* = 3 biologically independent experiments. (**B**) R-loop assay at 6 endogenous sites (R-loop 1–6) with dSaCas9 and corresponding sgRNAs performed to evaluate Cas9-independent off-target C-to-T conversion frequencies, with C-to-T editing frequency indicating the ratio of sequencing reads with C-to-T conversion. C positions showing the highest C-to-T activity at each R-loop site were shown, with two C positions in R-loop 5 included. *n* = 3 biologically independent experiments. (**C**) On-target base editing efficiencies versus DNA off-target effects with R loop assay. On-target and R-loop off-target base editing efficiencies were calculated as the mean of the most edited base in six endogenous sites. BE3 and BE4 could be considered as positive control, which displayed obvious Cas9-independent off-target edits while nCas9 was negative control, which displayed no on-target and off-target base editing efficiencies. (**D, E**) Jitter plots for RNA off-target edits showing C-to-U modifications in RNA transcripts in HEK293T cells induced by N-YE1-BE. RNA off-target edits were quantified using HaplotypeCaller (D) or MuTect2 (E). *n* = 4 biologically independent experiments. (**F**) Scheme of experimental design for DNA damage analysis. (**G**) Quantification of γH2AX signaling in HEK293T cells transfected with nCas9, Cas9, BE3, BE4, N-R33A-BE, N-R33A-K34A-BE or N-YE1-BE without sgRNA expression. The percentage of γH2AX positive population within GFP positive cells is shown. *n* = 3 biologically independent experiments, ***P< 0.001 with two-tailed unpaired *t*-test. (**H**) 3D scatter diagram showing the on-target base editing efficiencies, DNA off-target effects with R loop assay and γH2AX accumulation for BE3, BE4, N-R33A-BE, N-R33A-K34A-BE and N-YE1-BE in red sphere, with nCas9 (pink sphere) as negative control. All values in (B) and (G) are presented as mean ± s.e.m.

Rat APOBEC1 (rA1) has been exploited to generate functional CBE BE3, with UGI tethered to nCas9, which displayed obvious DNA and RNA off-target effects. Improved CBE variants such as R33A-BE3, R33A-K34A-BE3 (6) and BE3-W90Y-R126E (hereafter YE1-BE3) (9–11,26) have been generated to reduce or eliminate DNA and RNA off-target effects. As demonstrated previously (32), free UGI could more fully inhibit UNG to improve the fidelity and efficiency of base editing, here we used freely expressed UGI separate from CBE constructs to generate N-R33A-BE, N-R33A-K34A-BE and N-YE1-BE for subsequent analysis, with BE3 and BE4 as control. We initially provided side-by-side on-target base editing comparison for BE3, BE4 and optimized variants N-R33A-BE, N-R33A-K34A-BE and N-YE1-BE at six endogenous sites. It was revealed that BE3 and BE4 mainly displayed editing scope at C3-C9, N-R33A-BE displayed editing scope at C4-C7 and N-YE1-BE displayed editing scope at C4-C8 with increased editing activity (Figure 1A, Supplementary Figure S1). However, N-R33A-K34A-BE just displayed obvious C-to-T base editing activity at TC_5_C (underlying for target cytosine) of EMX site (Figure 1A, Supplementary Figure S1), in agreement with previous studies (6). We further evaluated the CBE-specific Cas9-independent DNA off-target effects with R-loop assay at six orthogonal R-loops (10), and it was shown that BE3 and BE4 displayed obvious off-target editing ranged from 0.14% to 27.0% while N-R33A-BE, N-R33A-K34A-BE and N-YE1-BE displayed minimized off-target editing from 0.07% to 7.0%, 0.08% to 0.7% and 0.3% to 5.1% (Figure 1B), in agreement with previous studies showing that YE1, R33A and R33A-K34A could lead to minimization of Cas9-independent DNA off-target edits (6,10,11). Additionally, gRNA-dependent DNA off-target effects at two well-established endogenous sites H4 and EMX were evaluated (1). It was revealed that N-YE1-BE induced higher on-target and comparable off-target editing activities while N-R33A-BE induced comparable on-target and reduced off-target editing activities as compared to BE3 and BE4 (Supplementary Figure S2a). Therefore, N-YE1-BE represented the optimized rAPOBEC1-derived CBE variant displaying robust on-target C-to-T base editing activity and minimized Cas9-independent DNA off-target effects (Figure 1C).

As it has been demonstrated MuTect2 was a more sensitive tool for RNA base editing analysis, and could effectively detect RNA A-to-I edits with < 10% efficiency (18), we re-analyzed published RNA-seq data to evaluate C-to-U RNA edits for BE3, R33A-K34A-BE3 (6) and YE1-BE3 (9–11,26), with MuTect2. It was revealed that R33A-K34A-BE3 induced similar RNA C-to-U off-target edits to nCas9 (Supplementary Figure S2b). However, both BE3 and YE1-BE3 induced obviously increased RNA C-to-U off-target edits to nCas9 (average of approximately 22.7-fold and 7.4-fold, respectively, Supplementary Figure S2b, c). We then generated our data by co-transfecting N-YE1-BE and non-specific sgRNA into HEK293T cells for RNA off-target analysis using HaplotypeCaller and MuTect2 for comparison (18). In agreement with previous studies (11), HaplotypeCaller detected slightly increased RNA C-to-U off-target edits as compared to nCas9 (average of approximately 1.8-fold, Figure 1D). Nevertheless, MuTect2 detected more C-to-U RNA off-target edits than HaplotypeCaller (average of approximately 3.9-fold), and more C-to-U RNA off-target edits were induced by N-YE1-BE than nCas9 (average of approximately 2.5-fold, Figure 1E), confirming that N-YE1-BE retained detectable C-to-U editing activity at RNA level. Additionally, we analyzed the RNA C-to-U off-target edits of N-YE1-BE with two different strategies including freebayes (33) and varscan (34). Both strategies detected more C-to-U RNA off-target edits than HaplotypeCaller (Supplementary Figure S2d). Overlap analysis revealed that ∼50% C-to-U RNA off-target edits identified using MuTect2 could be confirmed by freebayes or/and varscan analysis (Supplementary Figure S2e). We further generated our data for RNA off-target analysis of BE3, BE4, N-R33A-BE and N-R33A-K34A-BE with MuTect2. It was revealed that BE3 and BE4 induced robustly increased RNA C-to-U off-target edits, N-R33A-BE induced slightly increased while N-R33A-K34A-BE induced comparable RNA C-to-U off-target edits to nCas9 (average of approximately 5.3-fold, 5.5-fold, 1.6-fold and 1-fold, respectively, Supplementary Figure S2f).

DSB-associated DNA damage risks could be detected and quantified by accumulation of γH2AX (22). To provide more accurate quantification for deaminase-induced DSB-associated DNA damage risks, we evaluated N-YE1-BE by flow cytometry in HEK293T cells, with nCas9 and Cas9 as a negative and positive control, respectively (Figure 1F). BE3, BE4, N-R33A-BE and N-R33A-K34A-BE were also included to provide a comprehensive comparison for rAPOBEC1-derived CBE variants. As cytidine deaminase alone could induce robust DSBs and accumulation of γH2AX, here we initially evaluated DSB-associated DNA damage risks without sgRNA co-expression (Supplementary Figure S3). It was revealed that Cas9 induced significantly increased γH2AX accumulation (average of 29.2%) than nCas9 (average of 14.3%, Figure 1G), in agreement with previous studies showing that Cas9 alone could induce robust DNA damage responses (24,25). Furthermore, N-YE1-BE, BE3, BE4 and N-R33A-BE also induced significantly increased γH2AX accumulation (average of 31.5%, 38.4%, 40.8% and 26.1% respectively) than nCas9 (Figure 1G), in agreement with previous studies showing that CBEs induced obvious DNA damage responses (22,23). N-R33A-K34A-BE, an optimized CBE variant with minimized DNA/RNA off-target edits, displayed slightly increased γH2AX accumulation to nCas9 (average of 19.1% and 14.3% respectively, Figure 1G). We further evaluated DSB-associated DNA damage risks induced by N-YE1-BE together with scrambled sgRNA (sgRNA-NC), EMX (sgRNA-EMX) or H4 (sgRNA-H4). It was revealed that N-YE1-BE and Cas9 induced significantly increased γH2AX accumulation while N-R33A-K34A-BE induced slightly increased γH2AX accumulation with all three sgRNAs as compared to nCas9 (Supplementary Figure S4), indicating that sgRNA expression had little impact on γH2AX accumulation induced by N-YE1-BE. Therefore, DSB-associated DNA damage evaluation for CBEs in subsequent experiments was performed without sgRNA. Taken together, our analysis revealed that previously engineered CBE variant N-YE1-BE still displayed unnegligible safety risks, including slightly increased C-to-U RNA off-target edits and obvious DSB-associated DNA damage risks (Figure 1H), and further engineering of CBEs are required to ameliorate these safety risks.

Several groups and our studies demonstrated that TadA deaminases could be reprogrammed to generate improved CBEs with minimized off-target risks (35–39). Here we selected two representative TadA-derived CBEs TadCBEd and CBET1.46 for systematically analysis. It was revealed that TadCBEd and CBET1.46 displayed wider editing scope at C3-C11 with comparable editing activity to N-YE1-BE (Supplementary Figure S5a, b). R-loop analysis showed that TadCBEd displayed obvious off-target editing ranged from 2.78% to 33.2% while CBET1.46 displayed 0.69% to 11.8% off-target editing, comparable to N-YE1-BE (Supplementary Figure S5c-d). RNA off-target analysis revealed that TadCBEd and CBET1.46 induced slightly increased RNA C-to-U off-target edits to nCas9 (average of approximately 2.6-fold and 1.4-fold, respectively, Supplementary Figure S5e). Then γH2AX-dependent DNA damage analysis showed that TadCBEd and CBET1.46 induced significantly increased γH2AX accumulation (average of 33.3% and 25.6%, respectively) than nCas9 (average of 14.3%, Supplementary Figure S5f, g). We also evaluated the residual A-to-G DNA editing activity and gRNA-dependent DNA off-target effects for TadCBEd and CBET1.46. It was revealed that TadCBEd and CBET1.46 displayed > 10% A-to-G DNA editing activity at 3/6 endogenous sites (an average of 29–67% and 15–45%, respectively, Supplementary Figure S6a), and induced increased gRNA-dependent DNA off-target editing at two well-established endogenous sites H4 and EMX (Supplementary Figure S6b).

In summary, these analyses demonstrated that TadA-derived CBEs would possibly display detectable RNA off-target edits and DNA damage risks (Supplementary Figure S5g), indicating that TadA reprogramming did not necessarily generate CBEs with optimized performance. Therefore, further engineering is still required for additional optimization.

### CBE variants with refined scopes and minimized safety risks via LjCDA1 engineering

Lamprey cytidine deaminases (hereafter CDAs) are alternative cytidine deaminases that have been exploited to generate functional CBEs (2,13). However, safety risks of CDA-derived CBEs have not been evaluated systematically in previous studies. Here we initially evaluated their Cas9-independent DNA off-target editing risks with sanger sequencing for CDA-derived CBEs (detailed sequences in (13) and Supplementary Table S2) with R-loop assay (10), and revealed that LjCDA1- (also known as N-7-BE, hereafter N-Lj-BE) and LjCDA1L2_1- (also known as N-12-BE) derived CBEs displayed the lowest Cas9-independent DNA off-target edits (Supplementary Figure S7a). LjCDA1 was then chosen for subsequent protein engineering to refine the editing signatures. It has been demonstrated that two recognition loop regions (RL1 and RL2) existed in AID/APOBEC family proteins that are critical for ssDNA binding and sequence preferences (Supplementary Figure S7b) (40–43). We then replaced corresponding regions of LjCDA1 with RL1 or RL2 from different AID/APOBEC enzymes for CBE engineering, and the nomenclature of engineered LjCDA1 mutants was mainly as Lj-RL1/2 (X), with RL1/2 (X) representing the replacement of RL1/2 region with corresponding sequences from X deaminase (Supplementary Figure S7b). Editing activities and Cas9-independent DNA off-target edits were initially evaluated and compared, and it was revealed that 7/8 LjCDA1-derived functional CBE variants displayed detectable on-target editing activities and 8/8 displayed low Cas9-independent DNA off-target effects (Supplementary Figure S7c, d).

**Figure 2.**
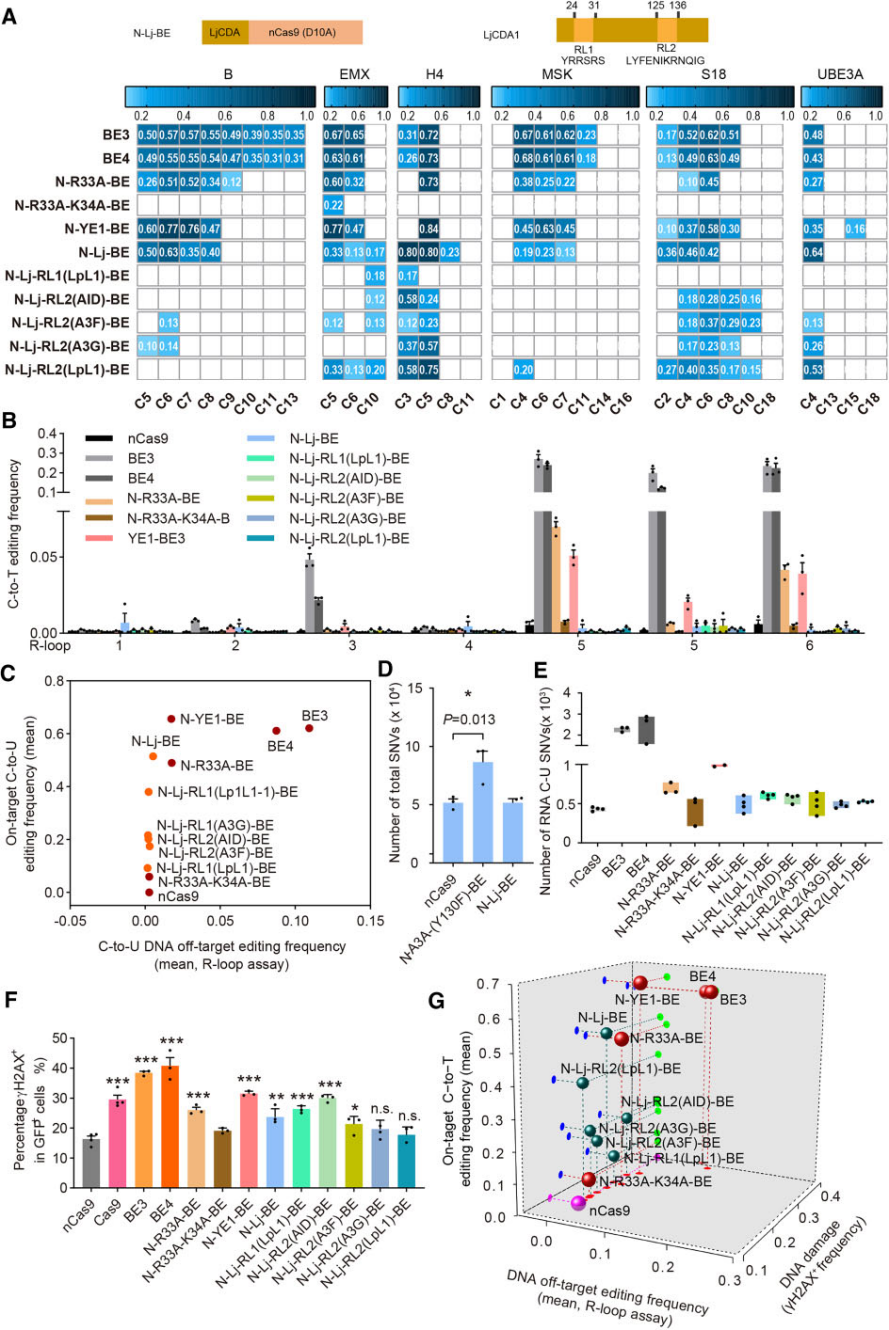
Editing signature, DNA/RNA off-target edits and DSB-associated DNA damage risks induced by representative LjCDA1-derived CBEs. (**A**) Little comics illustrating the architectures of LjCDA1-derived CBEs (above). Heatmaps showing on-target base editing efficiencies of representative LjCDA1-derived CBEs, including N-Lj-BE, N-Lj-RL1(LpL1)-BE, N-Lj-RL2(AID)-BE, N-Lj-RL2(A3F)-BE, N-Lj-RL2(A3G)-BE and N-Lj-RL2(LpL1)-BE, across six sgRNAs (detailed sequences in Supplementary Table S1) in HEK293T cells in blue gradient color, with BE3, BE4, N-R33A-BE, N-R33A-K34A-BE and N-YE1-BE as control. *n* = 3 biologically independent experiments. (**B**) R-loop assay at 6 endogenous sites (R-loop 1–6) with dSaCas9 and corresponding sgRNAs performed to evaluate Cas9-independent off-target C-to-T conversion frequencies, with C-to-T editing frequency indicating the ratio of sequencing reads with C-to-T conversion. C positions showing the highest C-to-T activity at each R-loop site were shown, with two C positions in R-loop 5 included. *n* = 3 biologically independent experiments. (**C**) On-target base editing efficiencies versus DNA off-target effects with R loop assay. On-target and R-loop off-target base editing efficiencies were calculated as the mean of the most edited base in six endogenous sites. The rAPOBEC1-derived CBEs and nCas9 were shown in red while LjCDA1-derived CBEs in orange. (**D**) Total number of all SNVs relative to the parent sample (HEK293T cells without any treatment, served as background) detected by WGS. The A3A-dervied CBE N-A3A-(Y130F)-BE were included as positive control. *n* = 3 biologically independent experiments, *P< 0.05 with two-tailed unpaired *t*-test. (**E**) Box plot showing the number of RNA C-to-U edits induced by representative LjCDA1-derived CBEs. BE3, BE4, N-R33A-BE, N-R33A-K34A-BE, N-YE1-BE and nCas9 were included as control. *n* = 3 or 4 biologically independent experiments. (**F**) Quantification of γH2AX signaling in HEK293T cells transfected with nCas9, Cas9, BE3, BE4, N-R33A-BE, N-R33A-K34A-BE, N-YE1-BE or representative LjCDA1-derived CBEs. The percentage of γH2AX positive population within GFP positive cells is shown. *n* = 3 or 4 biologically independent experiments, n.s.= no significance, *P< 0.05, **P< 0.01, ***P< 0.001 with two-tailed unpaired *t*-test. (**G**) 3D scatter diagram showing the on-target base editing efficiencies, DNA off-target effects with R loop assay and γH2AX accumulation for LjCDA1-derived CBEs (green sphere), with rA1-derived CBEs (red sphere) and nCas9 (pink sphere) as control. All values in (B), (D), (E) and (F) are presented as mean ± s.e.m.

As above screening results for LjCDA1-derived CBEs were quantified using sanger sequencing and still preliminary, six LjCDA1-derived CBEs were then selected for assessment of editing signatures and Cas9-independent DNA off-target edits with high-throughput sequencing. On-target base editing analysis revealed N-Lj-BE displayed editing scope at C2–C7 (the PAM was counted as positions 21–23 unless otherwise stated) with comparable editing activity to N-YE1-BE, and significantly higher than N-R33A-K34A-BE (Figure 2A, Supplementary Figure S8a, b). N-Lj-BE preferred CT > CA/CC>>CG (underline for target, Supplementary Figure S8c), in agreement with previous results (13). RL1 or RL2 replacement led to editing scope and activity changes. For example, N-Lj-RL1 (LpL1)-BE, with RL1 region grafted from LpCDA1L1_1, displayed significantly lower editing activity with editing scope precisely at C3 (Figure 2A, Supplementary Figure S8b); N-Lj-RL2 (AID)-BE, with RL2 region grafted from AID, displayed relative high activity with precise editing scope at C3 and preferred CA/CT > CC/CG & AC>>CC/GC/TC; N-Lj-RL2 (A3F)-BE, with RL2 region grafted from APOBEC3F, displayed significantly lower editing activity with editing scope at C4–C10 and preferred CT/CA > CC>>CG & AC>>CC/GC/TC; N-Lj-RL2 (A3G)-BE, with RL2 region grafted from APOBEC3G, displayed modest editing activity with editing scope at C3–C5 and preferred CT > CA > CC>>CG & AC > CC/GC > TC; N-Lj-RL2 (LpL1)-BE, with RL2 region grafted from LpCDA1L1_1, displayed relative high editing activity with editing scope at C3-C5 and preferred CT > CA > CC>>CG & AC > CC/GC/TC (Figure 2A, Supplementary Figure S8b, c). We noticed that RL1 or RL2 replacement also led to obvious changes of sequence preferences for corresponding CBEs. However, the changes of sequence preference just showed weak correlation to AID/APOBEC enzymes from which RL1 or RL2 was derived, indicating that the determination of sequence preferences was complicated and further analysis is required in the future to elucidate underling mechanisms. Furthermore, it has been shown that 3′ nucleotide had little impact on editing efficiency of CBEs (44–46), while here we found that LjCDA1-derived CBEs displayed preference for CT/CA and anti-preference for CG. This discrepancy might be attributed to the small sample size in this study and further analysis at more endogenous sites would provide comprehensive insights into the impact of 3′ nucleotide on editing efficiency. Additionally, R-loop assay with high-throughput sequencing confirmed that all representative LjCDA1-derived CBEs displayed significantly lower Cas9-independent DNA off-target edits than N-YE1-BE (Figure 2B). Combination of editing activity and R-loop assay analysis revealed that N-Lj-BE displayed robust on-target C-to-T base editing activity and minimized Cas9-independent DNA off-target effects (Figure 2C). Additionally, gRNA-dependent DNA off-target effects at H4 and EMX were also evaluated. It was revealed that most LjCDA1-derived CBEs induced significantly lower off-target editing activities than N-YE1-BE (Supplementary Figure S9a). We further performed whole-genome sequencing (WGS) for N-Lj-BE (Supplementary Figure S10), and revealed that N-Lj-BE produced similar C-to-T and total SNVs to nCas9 and significantly lower than A3A-derived CBEs N-A3A- (Y130F)-BE, which displayed obvious Cas9 independent DNA off-target edits (Figure 2D, Supplementary Figure S9b). Overall, both R-loop assay and WGS analysis demonstrated that LjCDA1-derived CBEs displayed minimized Cas9-indendepnt DNA off-target edits.

Then these six LjCDA1-derived CBEs were further analysed with MuTect2 to evaluate C-to-U RNA off-target editing risks. It was revealed that all six LjCDA1-derived CBEs induced similar C-to-U RNA off-target SNVs to nCas9 and significantly fewer SNVs than N-YE1-BE, indicating that LjCDA1-derived CBEs displayed minimized RNA off-target editing risks (Figure 2E).

Furthermore, DSB-associated DNA damage analysis revealed that LjCDA1-derived CBEs induced different DSB-associated DNA damage risks depending on LjCDA1 mutation types (Supplementary Figure S11), with N-Lj-RL1 (LpL1)-BE and N-Lj-RL2 (AID)-BE inducing significantly increased γH2AX signals (average of 26.3% and 30.1%, respectively), N-Lj-BE and N-Lj-RL2 (A3F)-BE inducing modestly increased γH2AX signals (average of 23.7% and 21.3%, respectively), while N-Lj-RL2 (A3G)-BE and N-Lj-RL2 (LpL1)-BE inducing similar γH2AX (average of 19.7% and 17.7%, respectively) to nCas9 (average of 16.3%) (Figure 2F).

Overall, here we demonstrated that LjCDA1-derived CBEs displayed minimal DNA and RNA off-target edits. DSB-associated DNA damage risks could also be eliminated through deaminase engineering to replace RL2 regions of LjCDA1, and derived CBEs displayed refined editing signatures, with N-Lj-RL2 (AID)-BE displaying editing scopes at single-base resolution. Nevertheless, in consideration of editing activity, R-loop assay analysis and DNA damage risks (Figure 2G), LjCDA1-derived CBEs did not achieve a combination of robust on-target editing activity, minimized DNA off-target editing effects and DNA damage risks, and further improvement is still required for the optimization of CBEs.

### Engineered CBEs with diversified scopes and minimized safety risks via APOBEC3A engineering in combined with internal fusion strategy

APOBEC3A (hereafter A3A) is another cytidine deaminase that has been engineered to generate functional CBEs (47). Though specific A3A mutations such as R128A and Y130F could reduce RNA off-target edits (9,23), Cas9-independent DNA off-target edits remained largely unresolved for A3A-derived CBE variants (10). Here we initially performed rational mutagenesis for A3A to minimize Cas9-independent DNA off-target edits. the nomenclature of engineered A3A mutants was mainly as A3A- (X) or A3A-X, with (X) representing point mutations while X without brackets representing fragment deletions or replacement. For example, A3A- (Y130F) and A3A- (N57G) (simplified as eA3A to stay the same with previous studies) were generated, as Y130F and N57G mutation led to obviously reduced RNA off-target edits (9,47). A3A- (D133G) was also included as neighboring Y132 was critical for RNA and single-stranded DNA (ssDNA) binding (48), A3A-STA13Del, truncated A3A variant with deletion of 13 amino acids at N-terminus, was generated to assess the impact of protein truncation on editing activity and off-target effects of A3A-derived CBEs. As recognition loop termed RL1 (amino acids GI) in A3A was involved in substrate specificity, A3A-GI-Del, an RL1 deleting variant, and A3A-RL1, RL1 replacement with APOBEC3G RL1 (amino acids EPWVR), which would lead to changes of sequence preference (43), were generated. Additionally, RL1 replacement was further introduced into eA3A and A3A- (D133G) to generate eA3A-RL1 and A3A- (D133G)-RL1 variants. Then potential CBEs were generated with engineered A3A mutants described above through either N- or C-terminal fusion strategies (Supplementary Figure S12a), for on-target editing activity and Cas9-independent DNA off-target screening (Supplementary Figure S12b, c). Initial screening with sanger sequencing showed that CBE variant N-eA3A-RL1-BE displayed robust C-to-T editing activity and the lowest Cas9-independent DNA off-target edits (Supplementary Figure S12b, c). Furthermore, various eA3A-RL1-derived CBEs were generated through internal fusion strategy, as internal fusion strategy could lead to diversified editing scopes and reduced RNA/DNA off-target edits (15–17). To exclude potential steric hindrance on deaminase activity, we designed two eA3A-RL1 forms, without or with additional 5′-NLS and 3′-flexible linker sequences (hereafter NL-eA3A-RL1), for CBE engineering (Supplementary Figure S13a). On-target base editing analysis showed that the editing scopes of eA3A-RL1-derived functional CBE variants were diversified by internal fusion strategy, and R-loop assay showed that the majority of eA3A-RL1-derived functional CBE variants retained low Cas9-independent DNA off-target edits (Supplementary Figure S13b, c). Furthermore, CBEs containing NL-eA3A-RL1, which is speculated to possess more flexible conformation than eA3A-RL1, generally displayed higher editing activities and slightly increased Cas9-independent DNA off-target edits at specific fusion sites such as residue 1249 (Supplementary Figure S13d, e).

**Figure 3.**
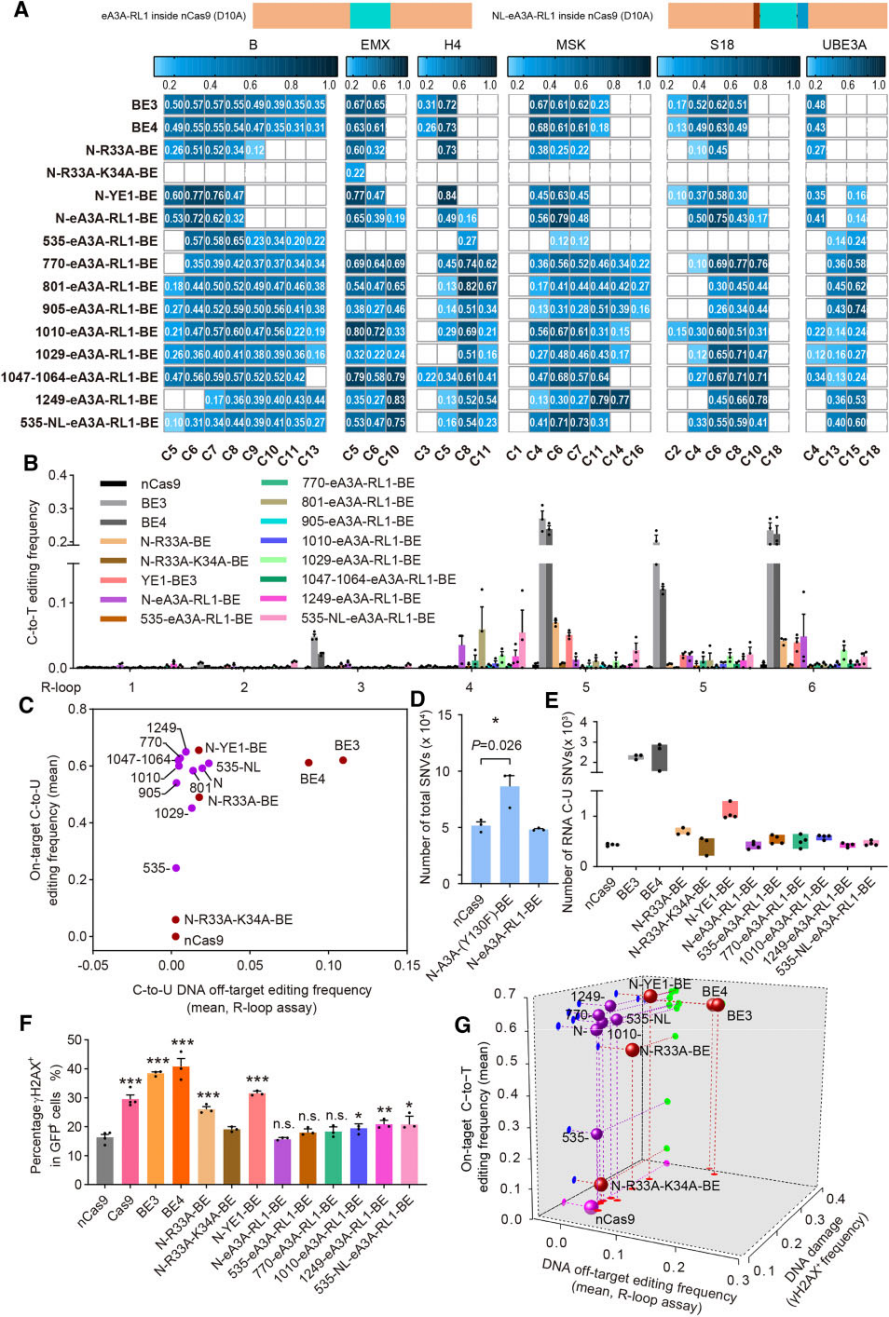
Editing signature, DNA/RNA off-target edits and DSB-associated DNA damage risks induced by representative eA3A-RL1-derived CBEs. (**A**) Little comics illustrating the architectures of eA3A-RL1-derived CBEs (above). Heatmaps showing on-target base editing efficiencies of representative eA3A-RL1-derived CBEs across six sgRNAs (detailed sequences in Supplementary Table S1) in HEK293T cells in blue gradient color, with BE3, BE4, N-R33A-BE, N-R33A-K34A-BE and N-YE1-BE as control. *n* = 3 biologically independent experiments. (**B**) R-loop assay at six endogenous sites (R-loop 1–6) with dSaCas9 and corresponding sgRNAs performed to evaluate Cas9-independent off-target C-to-T conversion frequencies, with C-to-T editing frequency indicating the ratio of sequencing reads with C-to-T conversion. C positions showing the highest C-to-T activity at each R-loop site were shown, with two C positions in R-loop 5 included. *n* = 3 biologically independent experiments. (**C**) On-target base editing efficiencies vs DNA off-target effects with R loop assay. On-target and R-loop off-target base editing efficiencies were calculated as the mean of the most edited base in six endogenous sites. The rAPOBEC1-derived CBEs and nCas9 were shown in red while representative eA3A-RL1-derived CBEs in purple. (**D**) Total number of all SNVs relative to the parent sample (HEK293T cells without any treatment, served as background) detected by WGS for N-eA3A-RL1-BE. The A3A-dervied CBE N-A3A-(Y130F)-BE were included as positive control. *n* = 3 biologically independent experiments, *P< 0.05 with two-tailed unpaired *t*-test. (**E**) Box plot showing the number of RNA C-to-U edits induced by representative eA3A-RL1-derived CBEs. N-YE1-BE and nCas9 were included as control. *n* = 3 or 4 biologically independent experiments. (**F**) Quantification of γH2AX signalling in HEK293T cells transfected with nCas9, Cas9, BE3, BE4, N-R33A-BE, N-R33A-K34A-BE, N-YE1-BE or representative eA3A-RL1-derived CBEs. The percentage of γH2AX positive population within GFP positive cells is shown. *n* = 3 or 4 biologically independent experiments, n.s.= no significance, *P< 0.05, **P< 0.01, ***P< 0.001. (**G**) 3D scatter diagram showing the on-target base editing efficiencies, DNA off-target effects with R loop assay and γH2AX accumulation for representative eA3A-RL1-derived CBEs (purple sphere), with rAPOBEC1-derived CBEs (red sphere) and nCas9 (pink sphere) as control. All values in (B), (D), (E) and (F) are presented as mean ± s.e.m.

As above screening results for A3A-dervied CBEs were quantified using sanger sequencing and still preliminary, then ten eA3A-RL1-derived CBEs were selected for assessment of editing signatures and Cas9-independent DNA off-target edits with high-throughput sequencing. On-target base editing analysis revealed that N-eA3A-RL1-BE displayed editing scope at C4–C7, which is similar to classic CBE BE3 (Figure 3A, Supplementary Figure S14a, b). However, sequence preference analysis demonstrated that N-eA3A-RL1-BE displayed high editing activity for all NC and CN site, with only slightly preference for CC site (Supplementary Figure S14c), while BE3 displayed minimal editing activity at GC site (1,13). Additionally, internal fusion strategy further diversified editing scopes and sequence preferences. For example, 535-eA3A-RL1-BE displayed editing scope at C6-C9 and preferred CC and CC sites; 770-eA3A-RL1-BE, 801-eA3A-RL1-BE and 905-eA3A-RL1-BE displayed similar editing scopes at C5–C15 with different sequence preferences (770-eA3A-RL1-BE for CG & GC; 801-eA3A-RL1-BE for CG > CA/CC/CT & GC/CC/TC > AC; 905-eA3A-RL1-BE CC/TC > GC/AC); 1010-eA3A-RL1-BE displayed editing scope at C4–C10 and preferred CG > CC > CA > CT & TC > CC > GC > AC; 1029-eA3A-RL1-BE displayed editing scope at C6–C11 and no obvious sequence preference; 1047–1064-eA3A-RL1-BE displayed editing scope at C5–C11 with no obvious sequence preference; 1249-eA3A-RL1-BE displayed editing scope at C8–C15 with anti-preference for CC and CC sites; 535-NL-eA3A-RL1-BE displayed editing scope at C6–C10 and C15 and slightly preferred CG and GC sites (Figure 3A, Supplementary Figure S14a–c). We noticed that 535-eA3A-RL1-BE and its flexible version 535-NL-eA3A-RL1-BE displayed different editing activities, scopes and sequence preferences, indicating that linker insertion might be valuable to modulate editing signatures of different CBEs (Figure 3A, Supplementary Figure S14a–c). Additionally, R-loop assay with high-throughput sequencing confirmed that all representative CBEs displayed comparable and even lower Cas9-independent DNA off-target edits than N-YE1-BE, with N-eA3A-RL1-BE and 535-NL-eA3A-RL1-BE displaying the highest off-target editing (∼5% C-to-T conversion frequency) (Figure 3B). Combination of editing activity and R-loop assay analysis for eA3A-RL1-derived CBEs revealed that 8/10 eA3A-RL1-derived CBEs displayed comparable on-target C-to-T base editing activity to N-YE1-BE and minimized Cas9-independent DNA off-target effects (Figure 3C). Additionally, gRNA-dependent DNA off-target effects at H4 and EMX were evaluated also evaluated. It was revealed that most eA3A-RL1-derived CBEs induced comparable or lower off-target editing activities than N-YE1-BE (Supplementary Figure S15a). We further performed whole-genome sequencing (WGS) for representative CBEs N-eA3A-RL1-BE (Supplementary Figure S10). eA3A-RL1-derived CBEs produced similar C-to-T and total SNVs to nCas9, while N-A3A- (Y130F)-BE produced significantly increased C-to-T and total SNVs (Figure 3D, Supplementary Figure S15b). Overall, both R-loop assay and WGS analysis confirmed that eA3A-RL1-derived CBEs displayed minimized Cas9-indendepnt DNA off-target edits.

In consideration of base editing signatures and Cas9-independent DNA off-target editing status, six eA3A-RL1-derived CBEs, including N-eA3A-RL1-BE, 535-eA3A-RL1-BE, 770-eA3A-RL1-BE, 1010-eA3A-RL1-BE, 1249-eA3A-RL1-BE and 535-NL-eA3A-RL1-BE, were chosen for RNA off-target editing analysis with MuTect2. It was revealed that all six CBE variants induced similar C-to-U RNA off-target SNVs to nCas9, indicating that eA3A-RL1-derived CBEs displayed minimized RNA off-target editing risks eA3A-RL1 represented an improved deaminase mutant with eliminated RNA editing activity (Figure 3E).

DSB-associated DNA damage analysis revealed that all six represented eA3A-RL1-derived CBEs induced similar or slightly higher levels of γH2AX signals (average of 15.6%, 17.9%, 18.3%, 19.4%, 20.8% and 10.8%, respectively) to nCas9 (average of 16.3%) (Figure 3F, Supplementary Figure S16). Therefore, this result indicated that eA3A-RL1 represented an improved deaminase mutant with almost eliminated genotoxicity risks.

Overall, in consideration of editing activity, R-loop assay analysis and DNA damage risks (Figure 3G), most eA3A-RL1-derived CBEs could achieve a combination of robust on-target editing activity, minimized DNA off-target editing effects and DNA damage risks, providing improved CBE tools to expand their future applications.

## Discussion

DSB-associated DNA damage risks of cytidine deaminases and derived CBEs have been demonstrated previously (19,20,22,23), which raised serious safety concerns for therapeutic applications of DNA base editors, especially those requiring long-term expression of base editors (49), such as the case for the adeno-associated virus (AAV) system delivered therapeutic attempts being extensively explored in various animal models including primates (49–54). In addition to clinical applications, base editors have also been adapted for pooled screening and high-throughput functional analysis of single nucleotide variants (55–57), which generally requires stable expression of base editors. DSB-associated DNA damage risks might affect cellular sensitivity and interfere with data interpretation of related screens to a large extent. Evaluation of DSB-associated DNA damage risks in previous studies was generally performed with Cas9 as negative control and γH2AX immunoblot or immunofluorescence for quantification. As Cas9 could induce obvious DNA damage risks and γH2AX accumulation (24,25), it is speculated that nCas9 would be better than Cas9 as negative control. Recently, DSB-associated DNA damage risks of CBEs were evaluated by γH2AX accumulation with flow cytometry and with deaminase-inactive CBEs as negative control, providing an updated platform for DSB-associated DNA damage quantification (22). It was also reported that DSB-associated DNA damage risks induced by CBEs could be reduced by cytidine deaminase splitting (22). However, direct comparison between Cas9 and nCas9 for DSB-associated DNA damage risks have not been performed previously, and intact CBEs with minimized DSB-associated DNA damage risks remain to be established. Here we provided a rational comparison for nCas9, Cas9 and standard CBEs. It was revealed that nCas9 was a more appropriate negative control, as Cas9 alone or with sgRNA co-expression did induce significantly increased γH2AX accumulation than nCas9. Though one study in mouse embryonic fibroblasts demonstrated that nCas9 generated similar γH2AX signal to Cas9, we noticed that authors used one specific sgRNA targeting B2 repeats with ∼350000 copies in the mouse genome, which might lead to more DSBs and increased γH2AX signal (58). Therefore, in combined with previous studies (22), we established an improved standards to evaluate deaminase-induced DNA damage risks by γH2AX accumulation quantification with flow cytometry and with nCas9 as negative control. We demonstrated that current deaminase engineering strategies to minimize DNA and RNA off-target edits did not necessarily eliminate DSB-associated DNA damage risks, as N-YE1-BE, an optimized BE3 variant with minimized DNA and RNA off-target edits, still induced robust DSB-associated DNA damage risks. In this study, through deaminase engineering of human APOBEC3A and lamprey LjCDA1, we developed several engineered CBE variants displaying minimized DSB-associated DNA damage risks as compared to nCas9, which represented the first successful attempts, to our knowledge, to reduce the DSB-associated DNA damage risks of constitutively active cytidine deaminase components.

It has been demonstrated that activation-induced cytidine deaminase (AID) generated genome-wide DSBs promiscuously without obvious target-site specificity (21). Here we also tried to mapping DSBs induced by nCas9, Cas9, N-YE1-BE or N-eA3A-RL1-BE alone by modified GUIDE-seq method without sgRNA co-expression. It was revealed that N-YE1-BE induced maximal number of 43964 DSBs, while Cas9 and nCas9 induced 20854 and 7803 DSBs, respectively (Supplementary Figure S17a). N-eA3A-RL1-BE, a representative CBE with minimized DNA damaging risks, induced 24248 DSBs, which is fewer than N-YE1-BE but more than Cas9 and nCas9 (Supplementary Figure S17a). As no obvious correlation was observed between DSB number and DNA damage risks for nCas9, Cas9, N-YE1-BE and N-eA3A-RL1-BE, additional factors such as DSB-induction activity might be also involved in the regulation of DSB-associated DNA damage risks. Additionally, we identified several representative regions with increased dsODNs integrations with N-YE1-BE but not N-eA3A-RL1-BE treatment (Supplementary Table S3). Then we evaluated the DSB-induction activity (Supplementary Figure S17b) at two representative regions identified with GUIDE-seq. At chr3 region, Cas9 displayed the highest DSB-induction activity, N-YE1-BE displayed lower but detectable DSB-induction activity while N-eA3A-RL1-BE and nCas9 displayed significantly lower DSB-induction activity. At chr16 region, only Cas9 and N-YE1-BE displayed detectable DSB-induction activity (Supplementary Figure S17c). Therefore, these results indicated that there are vulnerable regions in the genome to be targeted by N-YE1-BE but not engineered CBE N-eA3A-RL1-BE. Additionally, we also performed DSB induction activity analysis with CBEs in combined with sgRNA-1, and revealed that Cas9 displayed the highest DSB-induction activity, N-YE1-BE displayed lower but detectable DSB-induction activity while N-eA3A-RL1-BE and nCas9 rarely induced DSBs at on-target site (Supplementary Figure S17d), indicating that N-YE1-BE displayed higher DSB-induction activity when binding to targeted regions in the genome. Taken together, increased DNA damage risks induced by N-YE1-BE might be attributed to its binding ability to vulnerable regions in the genome and moderate DSB-induction activity as compared to Cas9. Minimized DNA damage risks of N-eA3A-RL1-BE might be attributed to reduced binding ability to vulnerable regions and minimization of DSB-induction activity.

In this study, we used freely expressed UGI separate from CBE constructs but not the classical constructs with UGI tethered to nCas9. The reasons could be classified as follows: Firstly, as we mainly tried to reduce or minimize the safety risks of cytidine deaminase domain in CBEs here, constructs without tethered UGI would provide more accurate DNA damage quantification for different CBEs as compared to nCas9 and Cas9. N-YE1-BE used in this study was also modified to exclude UGI region. Secondly, as demonstrated previously (32), free UGI could more fully inhibit UNG to improve the fidelity and efficiency of base editing, which would be valuable for therapeutic applications of CBEs. Nevertheless, overexpression of free UGI separately would not be suitable for therapeutic application. Recently, it was demonstrated that free UGI could be produced using construct with deaminase-nCas9-2A-UGI structure (35), which would display the combination of advantages of free UGI and structure simplicity. Though free UGI might increase Cas9-dependent off-target DNA edits as compared to classical constructs, such increased Cas9-dependent off-target DNA edits induced by free UGI might be minimized by other strategies, such as deaminase engineering, replacement of Cas9 with high-fidelity variants, or mRNA delivery to reduce existence time in cells. Thirdly, we also compared the DSB-associated DNA damage risks induced by N-YE1-BE (no UGI), YE1-BE3 (tethered UGI, classical construct) and N-YE1-2A-UGI (free UGI, modified construct), and it was revealed that the level of DNA damage risks induced by three constructs were as follows: N-YE1-BE > =YE1-BE3 > N-YE1-2A-UGI (Supplementary Figure S18). Therefore, free UGI represented a potential strategy that deserve systematically assessment in the future to evaluate its impact on safety risks of CBEs.

It's well-known that classic CBE BE3 induced robust DNA and RNA off-target edits and various strategies have been developed to minimize such safety risks. As the deaminase component of BE3 is rat APOBEC1, available engineered CBE variants with minimal off-target edits at both DNA and RNA levels were mainly derived from APOBEC1 mutants or orthologues (10,11,59). In addition, it should be noticed that commonly used HaplotypeCaller tool lacked sensitivity to detect RNA off-target edits with < 10% conversion frequency (18). In this study, RNA editing analysis with MuTect2 and HaplotypeCaller revealed that MuTect2 displayed significantly improved sensitivity to detect RNA off-target edits with <10% conversion frequency, while HaplotypeCaller rarely detected RNA off-target edits with < 10% conversion frequency. Therefore, MuTect2 could detect significantly more RNA off-target edits than HaplotypeCaller (Figure 1d-e), and represented a more sensitive tool for RNA off-target analysis. It was revealed that engineered BE3 variant N-YE1-BE still induced detectable RNA off-target edits. In this study, we generated a series of newly engineered CBEs, which contained mutated human APOBEC3A or lamprey LjCDA1 components, with minimal off-target edits at both DNA and RNA levels. Indeed, to our knowledge, this is the first time to generate APOBEC3A- and lamprey CDA-derived CBEs with minimized DNA and RNA off-target edits (Table 1). In addition, we further diversified the editing scopes and sequence preferences of engineered CBEs through internal fusion strategy and linker addition, which further expanded the toolkit of available CBEs with improved safety status. As previous studies have developed a series of CBEs with significantly reduced DNA and RNA off-target edits, here we selected representative engineered CBEs in this study and described previously to summarize their editing signatures and safety properties (Table 1). The majority of CBEs described in Table 1 displayed minimal DNA and RNA off-target editing risks. In addition to CBE variants described in this study, further genotoxicity evaluation and elimination for other engineered CBEs might provide more alternations for C-to-T substitutions with improved safety properties.

**Table 1.** Summary of editing signatures and safety risks for representative engineered CBEs

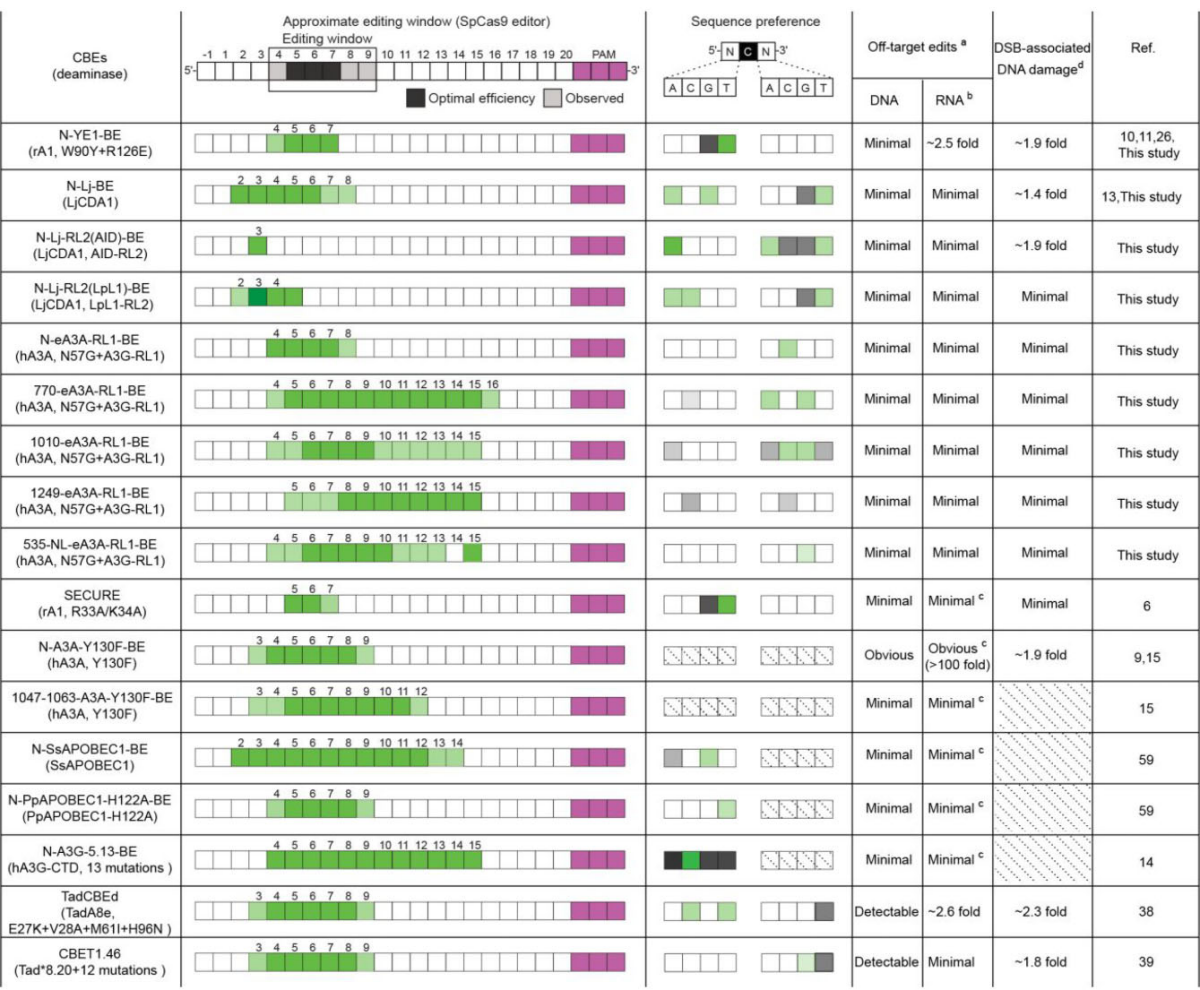

Editing signatures for representative engineered CBEs were summarized as previously. For sequence preference, dark/light green indicates strong/weak preferences while dark/light gray indicates strong/weak anti-preferences. ^a^Minimal indicates similar DNA or RNA off-target edits to background in Off-target edits column. ^b^RNA off-target edits were evaluated by MuTect2 unless otherwise stated. ^c^RNA off-target edits were evaluated by HaplotypeCaller. ^d^For DSB-associated DNA damage risks, nCas9 was used as control. Minimal indicates similar DSB-associated DNA damage risks to nCas9. Diagonal lines indicate lack of corresponding data.

Taken together, we developed a series of CBE tools with multidimensional safety improvement and diversified editing signatures. These newly engineered CBEs would be valuable for future applications in biomedical and clinical translational researches, especially for researches with stringent security requirement.

## Supplementary Material

gkad855_Supplemental_Files

## Data Availability

Raw high-throughput sequencing data are available in the NCBI sequence Read Archive database (PRJNA1006866, PRJNA1006518). All plasmids described in this work are available upon reasonable request. Other additional relevant data are available upon reasonable request.
