# Supplementary material for: Engineering of cytosine base editors with DNA damage minimization and editing scope diversification
Source: Nucleic Acids Res. 2023 Oct 16;51(20):e105. doi: 10.1093/nar/gkad855 (PMC10639057; doi:10.1093/nar/gkad855)
Supplement: gkad855_Supplemental_Files [file gkad855_supplemental_files.zip › Supplementary Information-2nd revision-0821.pdf]

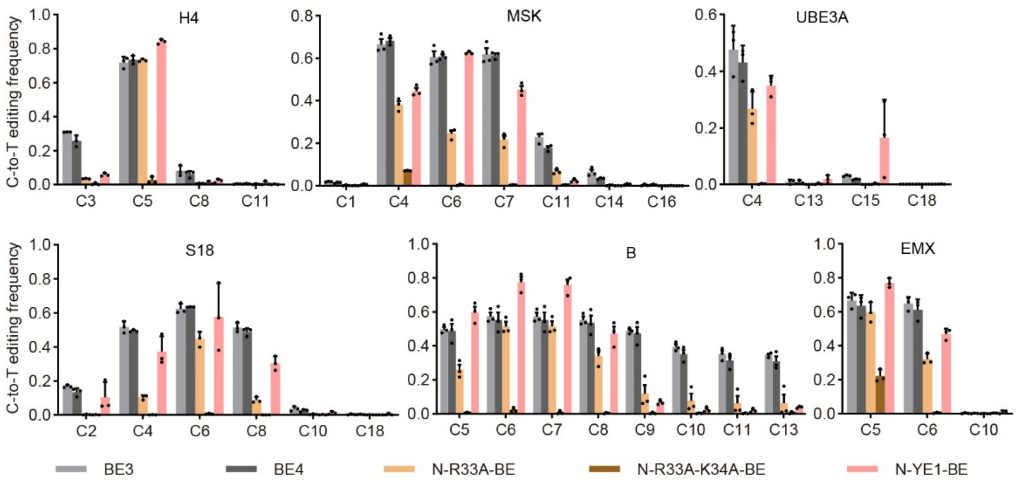

Supplementary Fig. 1 Overall editing percentage of edited alleles for representative rA1-derived CBEs. On-target base editing efficiencies of BE3, BE4, N-R33A- BE, N-R33A-K34A-BE and N-YE1-BE across 6 sgRNAs (detailed sequences in Supplementary Table 1) in HEK293T cells (positive cells were collected by flow cytometry). n=3 biologically independent experiments. All values are presented as mean ± s.e.m.

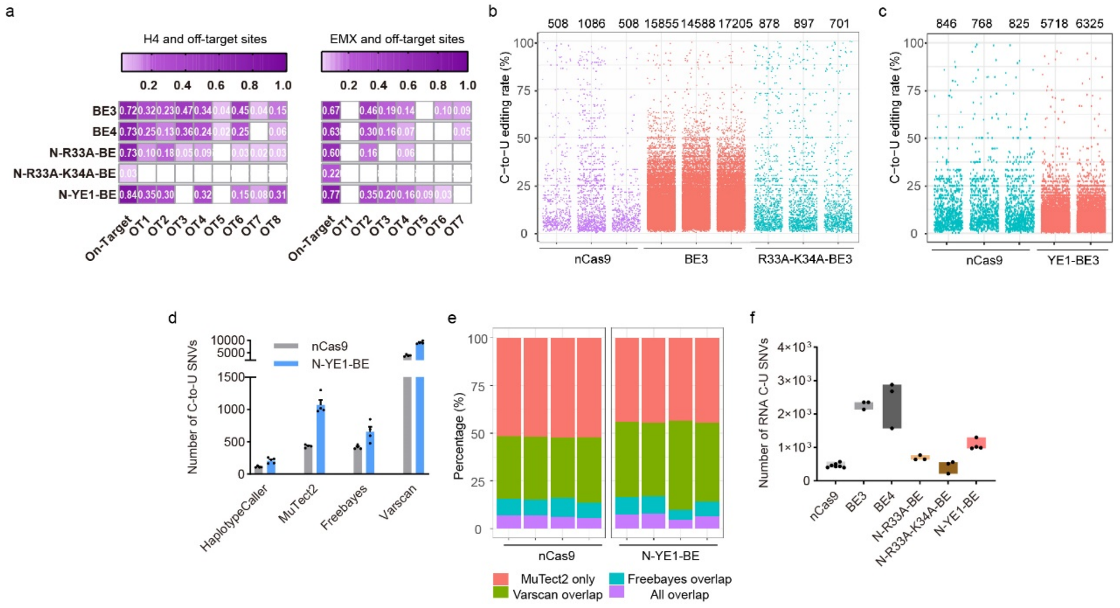

Supplementary Fig. 2 The gRNA-dependent DNA off-target effects and RNA off-target effects of representative rA1-derived CBEs. a, Heatmaps showing gRNA-dependent DNA off-target effects at two endogenous sites H4 and EMX for BE3, BE4, N-R33A-BE, N-R33A-K34A-BE and N-YE1-BE in purple gradient color. n=3 biologically independent experiments. **b-c,** Jitter plots for RNA off-target edits showing C-to-U modifications in RNA transcripts in HEK293T cells with RNA-seq data in published paper (6,9). RNA off-target edits were quantified using MuTect2 for induced by BE3 (b), K33A-R33A-BE3 (b) and YE1-BE3 (c). **d,** Box plot showing the number of RNA C-to-U edits in HEK293T cells induced by N-YE1-BE quantified using HaplotypeCaller, MuTect2, Freebayes and Varscan. n=4 biologically independent experiments. All values are presented as mean ± s.e.m. **e,** Overlap analysis for RNA C-to-U edits quantified by MuTect2, Freebayes and Varscan. **f,** Box plot showing the number of RNA C-to-U edits induced by BE3, BE4, N-R33A-BE, N-R33A-K34A-BE and N-YE1-BE, with nCas9 as control. n=3 or 4 biologically independent experiments.

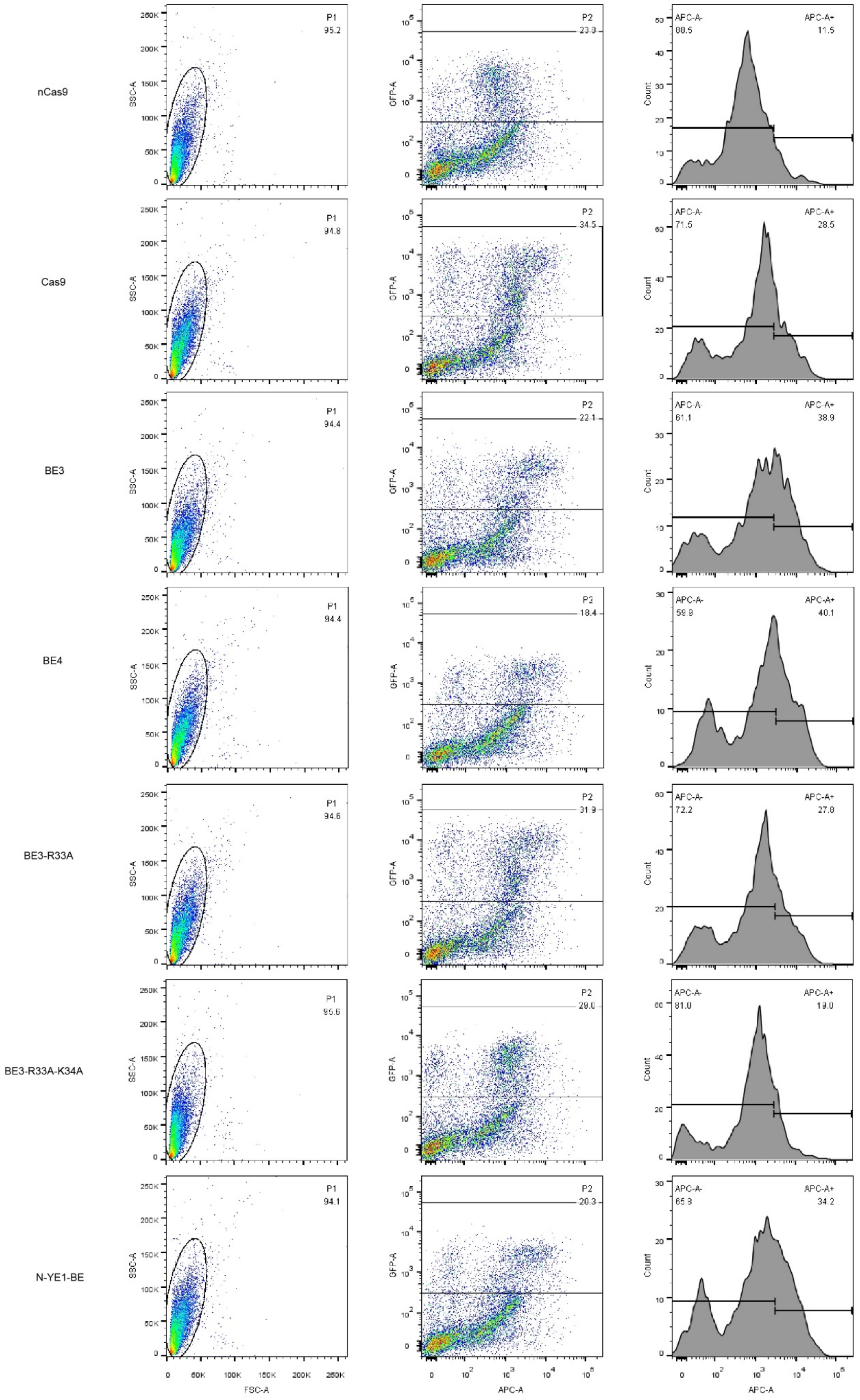
nCas9
P1 95.2
P2 23.8
APC-A- 88.5
APC-A+ 11.5
Cas9
P1 94.8
P2 34.5
APC-A- 71.5
APC-A+ 28.5
BE3
P1 94.4
P2 22.1
APC-A- 61.1
APC-A+ 38.9
BE4
P1 94.4
P2 18.4
APC-A- 59.9
APC-A+ 40.1
BE3-R33A
P1 94.6
P2 31.9
APC-A- 72.2
APC-A+ 27.8
BE3-R33A-K34A
P1 95.6
P2 29.0
APC-A- 81.0
APC-A+ 19.0
N-YE1-BE
P1 94.1
P2 20.3
APC-A- 65.8
APC-A+ 34.2
SSC-A
GFP-A
Count
FSC-A
APC-A
APC-A

Supplementary Fig. 3 Gating strategy of positive cells and γH2AX antibody fluorescence intensity for flow cytometry analysis. Viable cells were initially selected based on SSC-A and FSC-A plot. Then cells transfected with enzymes or CBEs were selected based on GFP intensity, while γH2AX antibody fluorescence intensity was analyzed for GFP-positive cells. Representative gating strategy plots for nCas9, Cas9, BE3, BE4, N-R33A-BE, N-R33A-K34A-BE and N-YE1-BE were shown. n=3 biologically independent experiments.

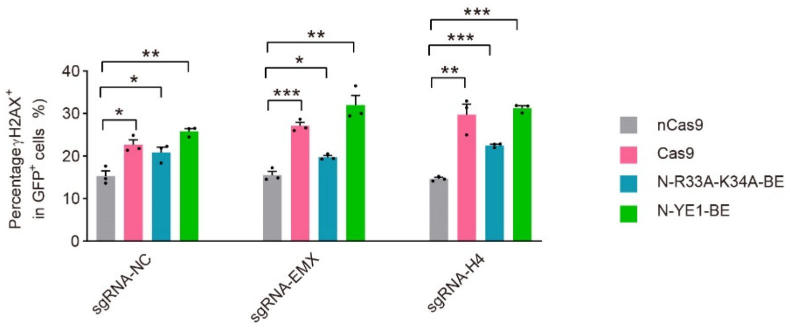

Supplementary Fig. 4 DSB-associated DNA damage analysis for rA1-derived CBEs co-expressing specific sgRNA. Quantification of γH2AX signaling in HEK293T cells transfected with nCas9, Cas9, N-R33A-K34A-BE or N-YE1-BE together with scrambled sgRNA (sgRNA-NC), sgRNA-EMX or sgRNA-H4. The percentage of γH2AX positive population within GFP positive cells is shown. n=4 biologically independent experiments, $*P<0.05$, $**P<0.01$, $***P<0.001$ with two-tailed unpaired t-test analysis. All values are presented as mean ± s.e.m.

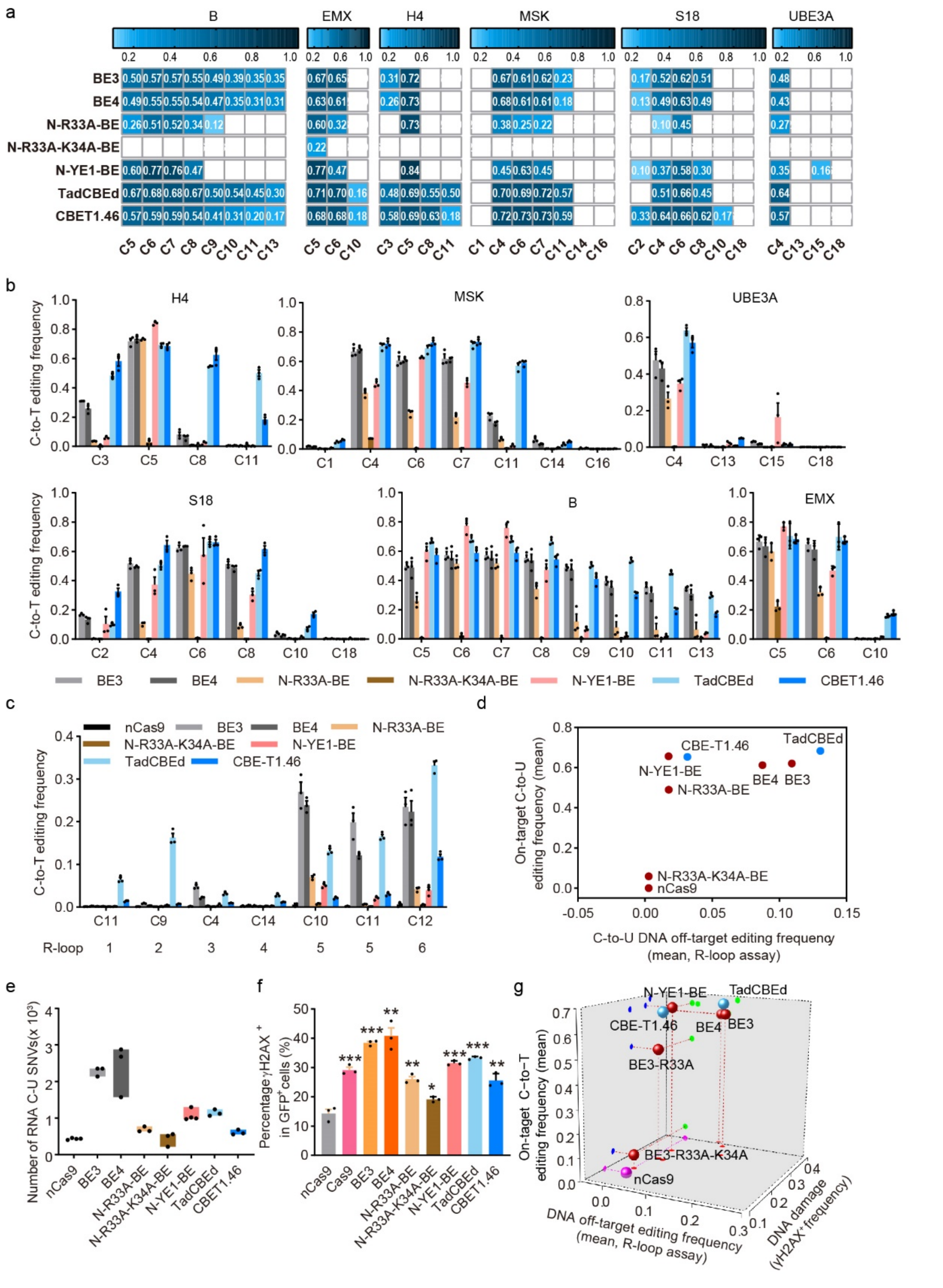

Supplementary Fig. 5: Editing signature, DNA/RNA off-target edits and DSB-associated DNA damage risks induced by representative TadA-derived CBEs. a-b. On-target base editing efficiencies of representative TadA-derived CBEs TadCBEd and CBET1.46, across 6 sgRNAs (detailed sequences in Supplementary Table 1) in HEK293T cells were shown in Heatmaps (**a**) and bar charts (**b**), with BE3, BE4, N-R33A-BE, N-R33A-K34A-BE and N-YE1-BE as control. n=3 biologically independent experiments. **c,** R-loop assay at 6 endogenous

sites (R-loop 1-6) with dSaCas9 and corresponding sgRNAs performed to evaluate Cas9-independent off-target C-to-T conversion frequencies, with C-to-T editing frequency indicating the ratio of sequencing reads with C-to-T conversion. C positions showing the highest C-to-T activity at each R-loop site were shown, with two C positions in R-loop 5 included. n=3 biologically independent experiments. **d,** On-target base editing efficiencies vs DNA off-target effects with R loop assay. On-target and R-loop off-target base editing efficiencies were calculated as the mean of the most edited base in 6 endogenous sites. The rAPOBEC1-derived CBEs and nCas9 were shown in red while TadA-derived CBEs in blue. **e,** Box plot showing the number of RNA C-to-U edits induced by representative TadA-derived CBEs. BE3, BE4, N-R33A-BE, N-R33A-K34A-BE, N-YE1-BE and nCas9 were included as control. n=3 or 4 biologically independent experiments. **f,** Quantification of γH2AX signaling in HEK293T cells transfected with nCas9, Cas9, BE3, BE4, N-R33A-BE, N-R33A-K34A-BE, N-YE1-BE or representative TadA-derived CBEs. The percentage of γH2AX positive population within GFP positive cells is shown. n=3 biologically independent experiments, n.s.= no significance, *P<0.05, **P<0.01, ***P<0.001 with two-tailed unpaired t-test. **g,** 3D scatter diagram showing the on-target base editing efficiencies, DNA off-target effects with R loop assay and γH2AX accumulation for TadA-derived CBEs (blue sphere), with rA1-derived CBEs (red sphere) and nCas9 (pink sphere) as control. All values in (**b**), (**c**), (**e**)and (**f**) are presented as mean ± s.e.m.

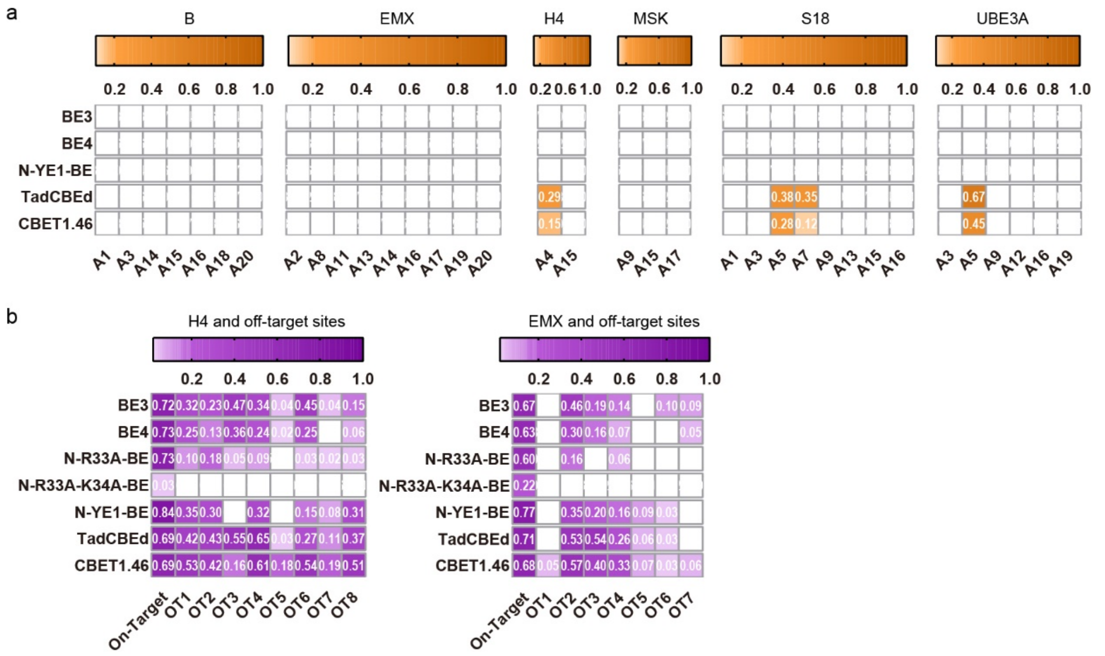

Supplementary Fig. 6 The residual A-to-G editing activity and gRNA-dependent DNA off-target effects of representative TadA-derived CBEs. a, Heatmaps showing on-target A-to-G base editing efficiencies of representative TadA-derived CBEs TadCBEd and CBET1.46, across 6 sgRNAs (detailed sequences in Supplementary Table 1) in HEK293T cells in orange gradient color, with BE3, BE4, N-R33A-BE, N-R33A-K34A-BE and N-YE1-BE as control. n=3 biologically independent experiments. **b,** Heatmaps showing gRNA-dependent DNA off-target effects at two endogenous sites H4 and EMX for TadA-derived CBEs, BE3, BE4, N-R33A-BE, N-R33A-K34A-BE and N-YE1-BE in purple gradient color. n=3 biologically independent experiments.

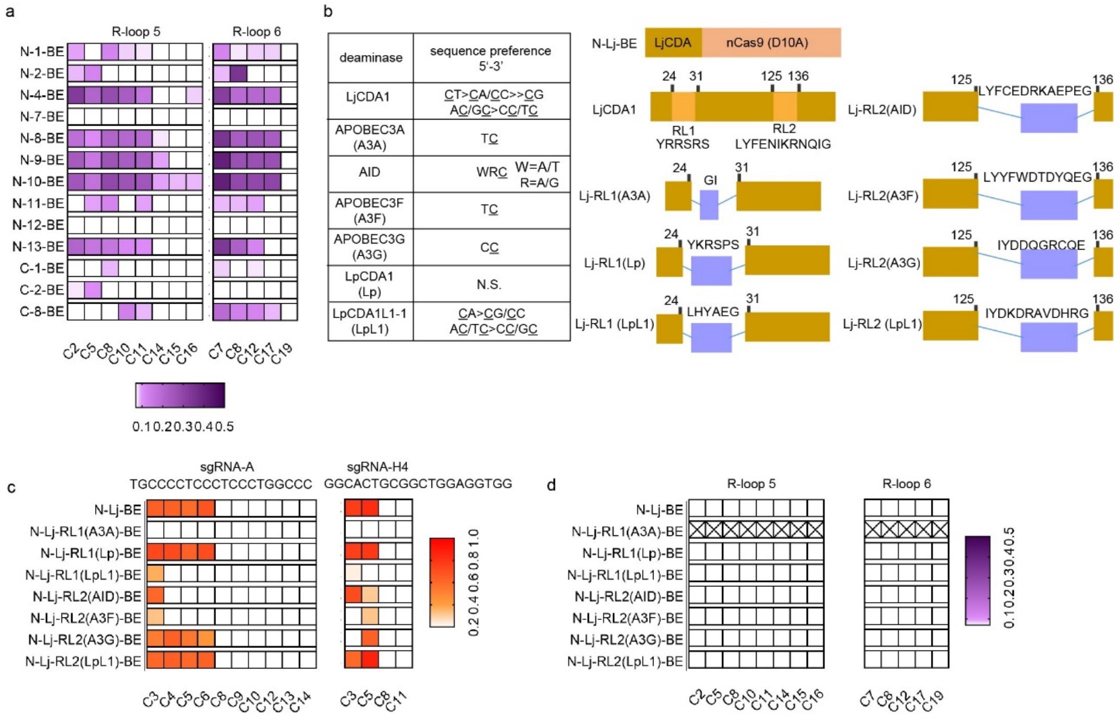

Supplementary Fig. 7 Editing activity and Cas9-independent off-target edits of CBEs derived from lamprey cytidine deaminases. a, Heatmaps showing R-loop assay screening against 2 endogenous sites (R-loop 5/6) with dSaCas9 and corresponding sgRNAs performed with sanger sequencing to evaluate Cas9-independent off-target C-to-T conversion frequencies of CBEs derived from lamprey cytidine deaminases (detailed sequences in Supplementary Table 2) in purple gradient color. **b,** Diagrams showing sequence preferences of cytidine deaminases used for protein engineering of LjCDA1 (left). Scheme of construction strategies for LjCDA1 engineering and nomenclature (right). Generally, RL1/2 (X) represents the replacement of RL1/2 region with corresponding sequences from X deaminase. **c,** Heatmaps showing screening of on-target base editing efficiencies for LjCDA1-derived CBEs in HEK293T cells against sgRNA-A and sgRNA-H4 in orange gradient color. **d,** Heatmaps showing R-loop assay against 2 endogenous sites (R-loop 5/6) with dSaCas9 and corresponding sgRNAs performed with sanger sequencing to evaluate Cas9-independent off-target C-to-T conversion frequencies of LjCDA1-derived CBEs in purple gradient color.

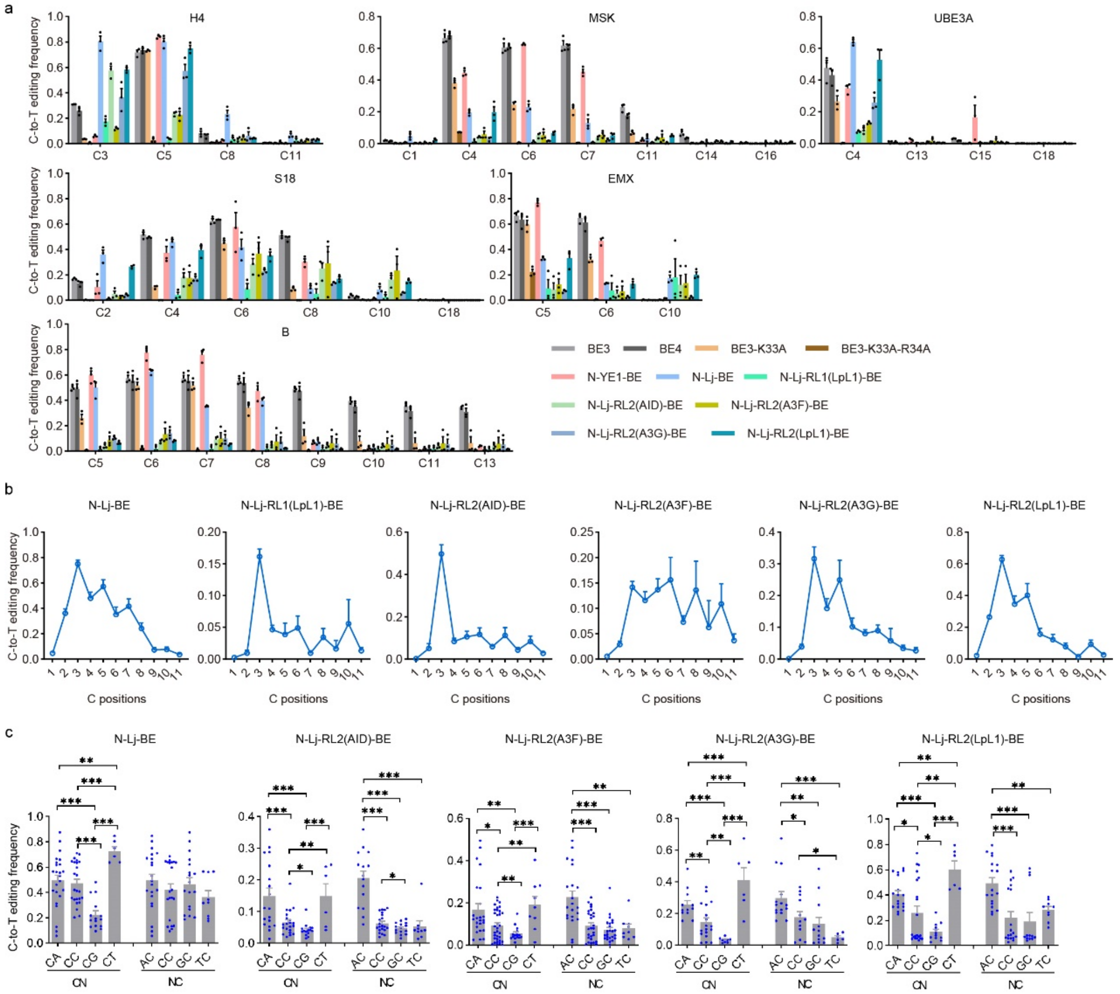

Supplementary Fig. 8 Editing scopes and sequence preferences of LjCDA1-derived CBEs. a, Overall editing percentage of edited alleles for representative LjCDA-derived CBEs across 6 sgRNAs (detailed sequences in Supplementary Table 1) in HEK293T cells (positive cells were collected by flow cytometry). n=3 biologically independent experiments. **b,** Editing scopes of representative LjCDA-derived CBEs across 6 endogenous sites. From left to right: N-Lj-BE, N-Lj-RL1(LpL1)-BE, N-Lj-RL2(AID)-BE, N-Lj-RL2(A3F)-BE, N-Lj-RL2(A3G)-BE and N-Lj-RL2(LpL1)-BE. n = 3 biologically independent experiments. **c,** sequence preferences of representative LjCDA1-derived CBEs. Generally, positions containing at least one C site with an average of >20% C-to-T editing frequency were included for sequence preference analysis. Then the impact of base types upstream or downstream of edited cytosine (NC or CN) was analyzed accordingly. **P*<0.05, ***P*<0.01, ****P*<0.001 with one-tailed unpaired t-test analysis. n = 3 biologically independent experiments. All values are presented as mean ± s.e.m.

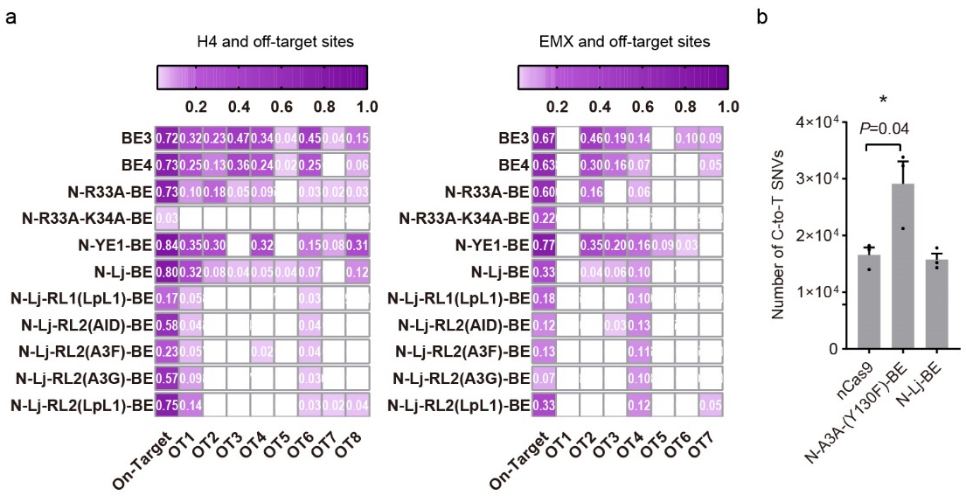

Supplementary Fig. 9 The gRNA-dependent DNA off-target effects and WGS analysis of representative LjCDA1-derived CBEs. a, Heatmaps showing gRNA-dependent DNA off-target effects at two endogenous sites H4 and EMX for BE3-no UGI, N-K33A-R34A-BE, N-YE1-BE and representative LjCDA1-derived CBEs in purple gradient color. n=3 biologically independent experiments. **b,** Total number of C-to-T (and G-to-A at complementary strand) SNVs relative to the parent sample detected by WGS. The A3A-dervied CBEs N-A3A-(Y130F)-BE were included as positive control. n=3 biologically independent experiments, *P<0.05 with two-tailed unpaired t-test. All values are presented as mean ± s.e.m.

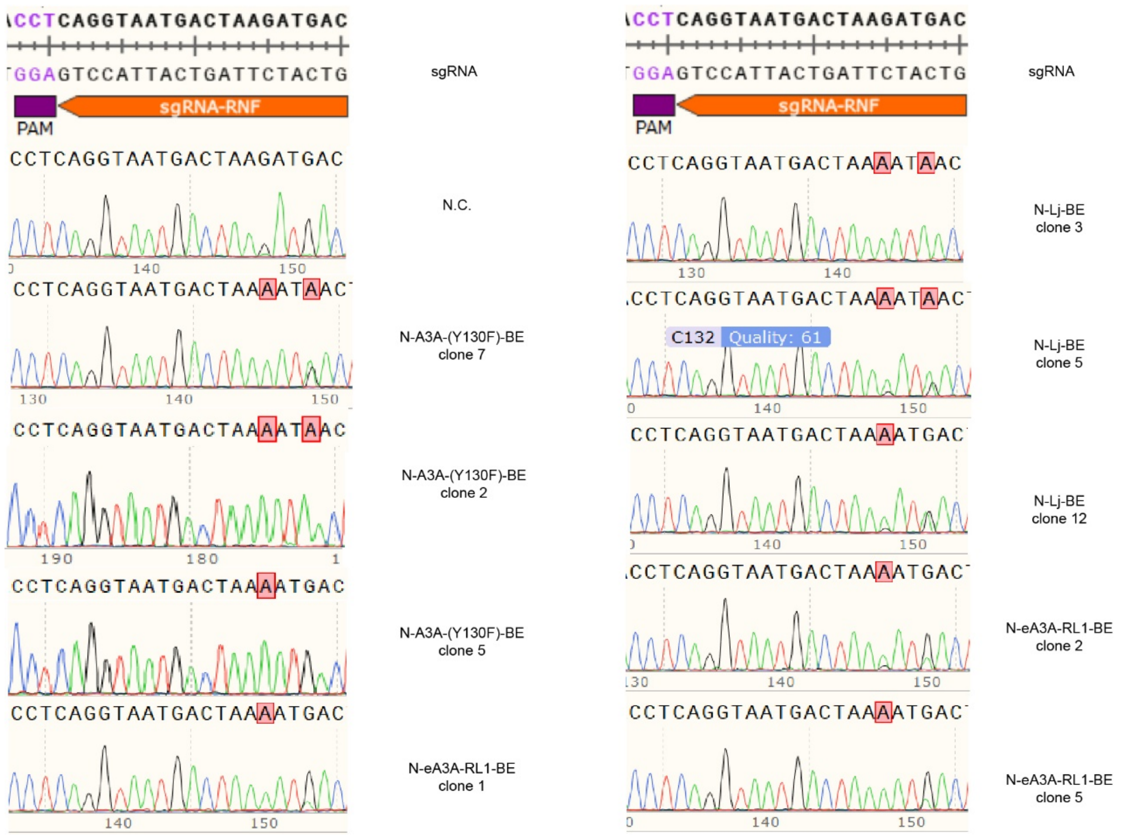

Supplementary Fig. 10 On-target base editing signature for cell colony used for whole genome sequencing analysis. HEK293T cells were transfected with indicated CBEs and sgRNA-RNF, which co-expressing puromycin resistance gene. Cells were then treated with puromycin to pick up single cell colony and cultured for another 4 weeks. During cell culture processes, part of cells was collected for targeted amplification and on-target base editing signature was assessed by sanger sequencing. Cell clones displaying on-target C-to-T editing were cultured for genome isolation and whole genome sequencing analysis to examine DNA off-target activities of representative CBEs.

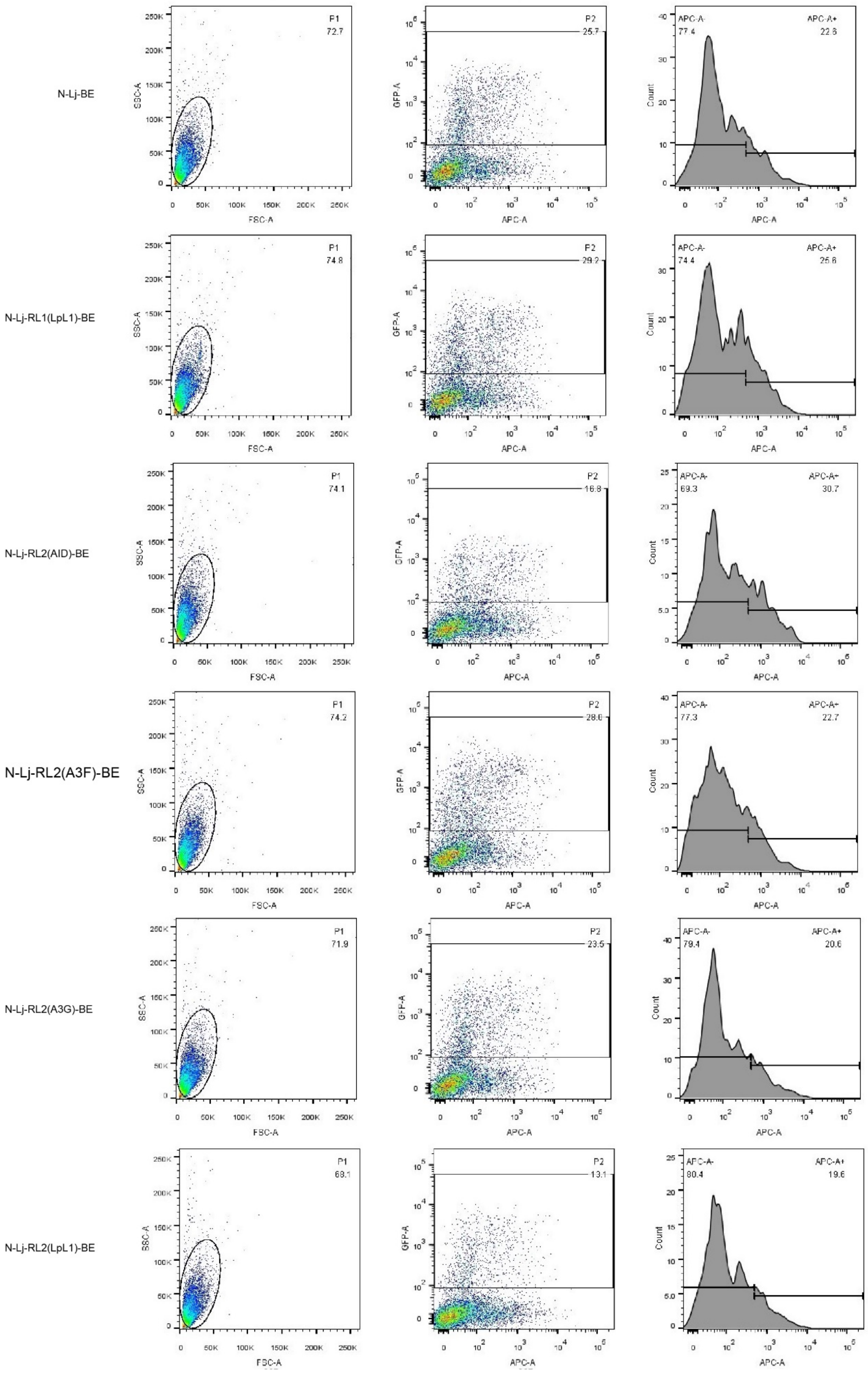

Supplementary Fig. 11 Gating strategy of positive cells and γH2AX antibody

fluorescence intensity for flow cytometry analysis. Viable cells were initially selected based on SSC-A and FSC-A plot. Then cells transfected with enzymes or CBEs were selected based on GFP intensity, while γH2AX antibody fluorescence intensity was analyzed for GFP-positive cells. Representative gating strategy plots for representative LjCDA1-derived CBEs were shown. n=4 biologically independent experiments.

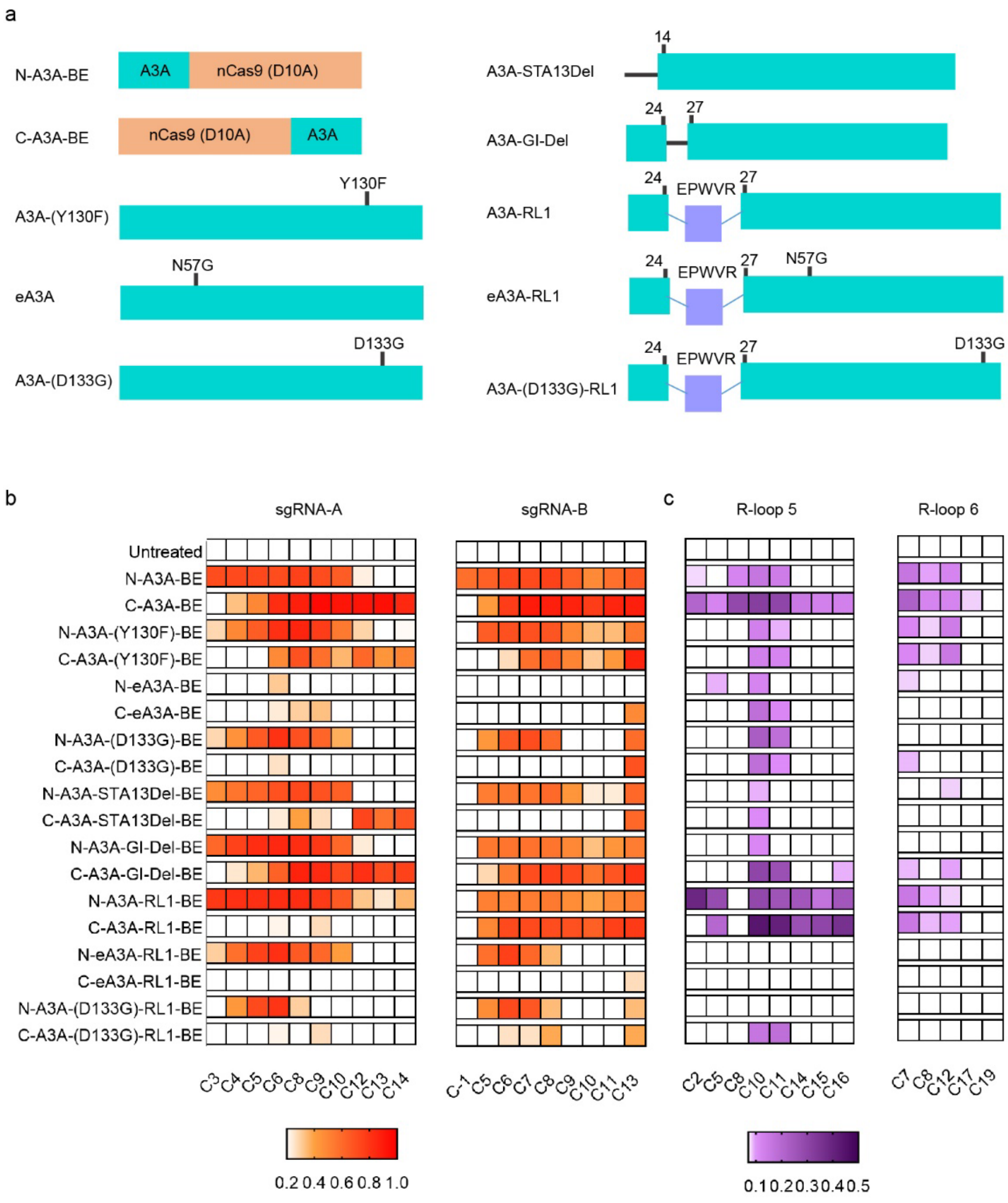

Supplementary Fig. 12 APOBEC3A engineering for CBE improvement, with assessment of editing activity and Cas9-independent off-target edits. a, Scheme of construction strategies for APOBEC3A (A3A) engineering and nomenclature (right). Generally, A3A-(X) represents A3A with specific mutation, with A3A-(N57G) simplified as eA3A as described previously(1). STA13Del represents deletion of 13 amino acids at N-terminus. GI-Del represents deletion of RL1 region. RL1 represents replacement of RL1 region with corresponding sequence from APOBEC3G (A3G). **b,** Heatmaps showing screening of on-target base editing efficiencies for engineered A3A-derived CBEs in HEK293T cells against sgRNA-A and sgRNA-B in orange gradient color. **c,** Heatmaps showing R-loop assay against 2

endogenous sites (R-loop 5/6) with dSaCas9 and corresponding sgRNAs performed with sanger sequencing to evaluate Cas9-independent off-target C-to-T conversion frequencies of engineered A3A-derived CBEs in purple gradient color.

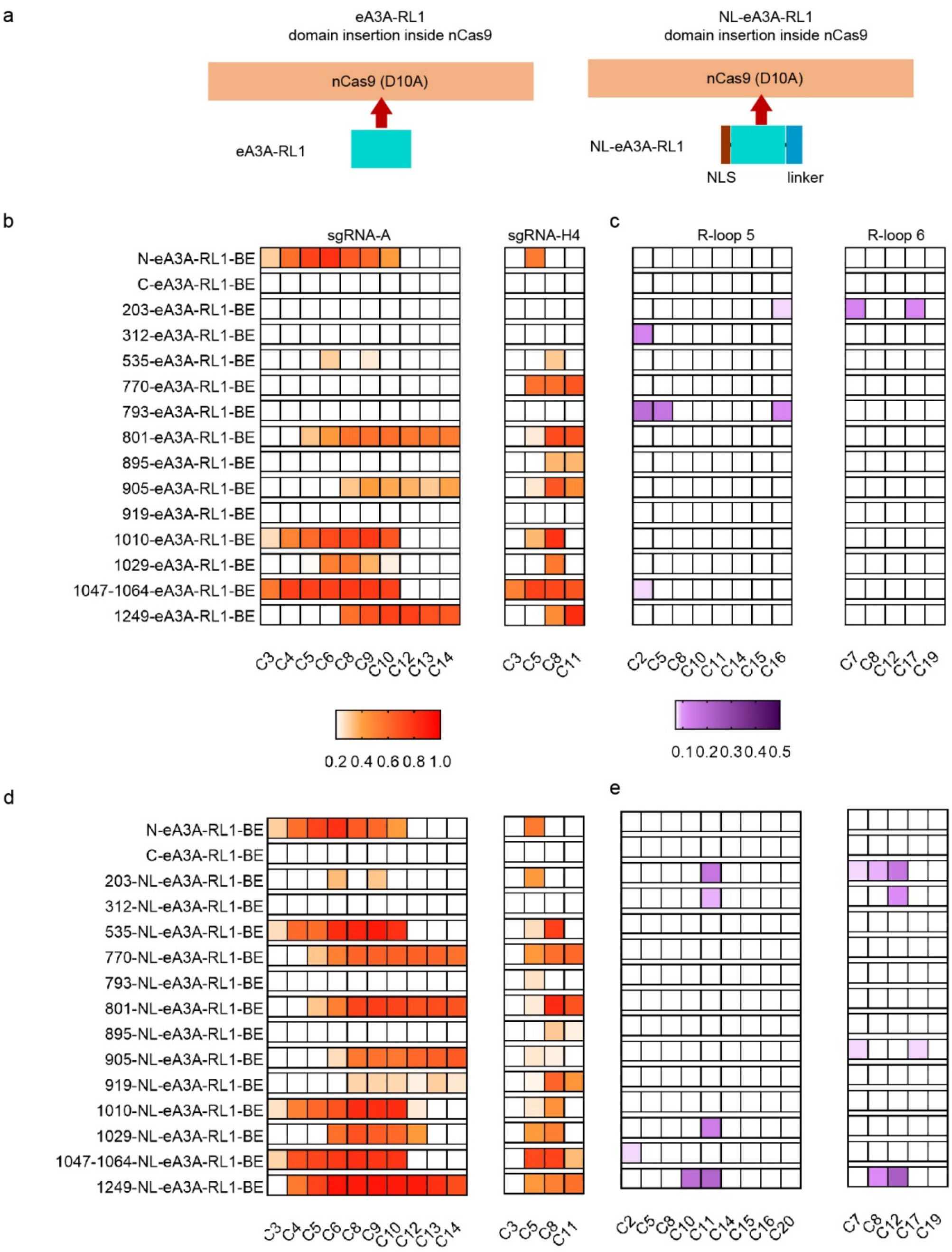

Supplementary Fig. 13 Editing activity and Cas9-independent off-target edits of eA3A-RL1-derived CBEs generated by eA3A-RL1 in combined with internal fusion strategy for CBE improvement. a, Schemes showing construction strategies for eA3A-RL1-derived CBEs with eA3A-RL1 insertion inside nCas9. NL-eA3A-RL1 represents eA3A-RL1 engineered deaminase containing 5’-NLS sequence and 3’-linker. **b,** Heatmaps showing screening of on-target base editing efficiencies for eA3A-RL1-derived CBEs in HEK293T cells against sgRNA-A and sgRNA-H4 in orange gradient color. **c,** Heatmaps showing R-loop assay against 2

endogenous sites (R-loop 5/6) with dSaCas9 and corresponding sgRNAs performed with sanger sequencing to evaluate Cas9-independent off-target C-to-T conversion frequencies of A3A-RL1-derived CBEs in purple gradient color. **d,** Heatmaps showing screening of on-target base editing efficiencies for NL-eA3A-RL1-derived CBEs in HEK293T cells against sgRNA-A and sgRNA-H4 in orange gradient color. **e,** Heatmaps showing R-loop assay against 2 endogenous sites (R-loop 5/6) with dSaCas9 and corresponding sgRNAs performed with sanger sequencing to evaluate Cas9-independent off-target C-to-T conversion frequencies of NL-A3A-RL1-derived CBEs in purple gradient color.

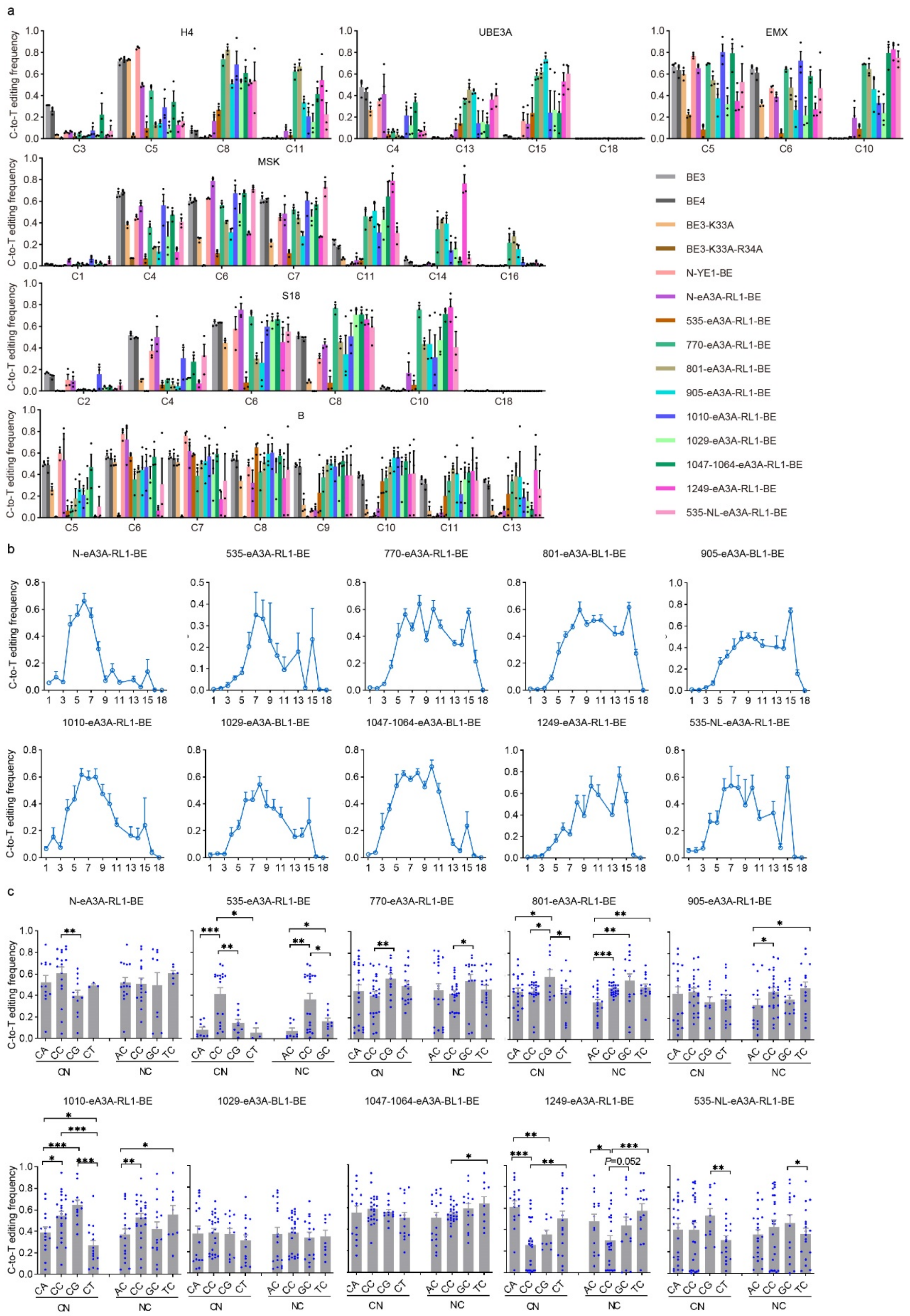

Supplementary Fig. 14 Editing scopes and sequence preferences of eA3A-RL1-derived CBEs. a, Overall editing percentage of edited alleles for representative eA3A-derived CBEs

across 6 sgRNAs (detailed sequences in Supplementary Table 1) in HEK293T cells (positive cells were collected by flow cytometry). n=3 biologically independent experiments. **b,** Editing scopes of representative eA3A-RL1-derived CBEs across 6 endogenous sites. n = 3 biologically independent experiments. All values are presented as mean ± s.e.m. **c,** sequence preferences of representative eA3A-RL1-derived CBEs. Generally, positions containing at least one C site with an average of >20% C-to-T editing frequency were included for sequence preference analysis. Then the impact of base types upstream or downstream of edited cytosine (NC or CN) was analyzed accordingly. *P<0.05, **P<0.01, ***P<0.001 with one-tailed unpaired t-test analysis. n = 3 biologically independent experiments. All values are presented as mean ± s.e.m.

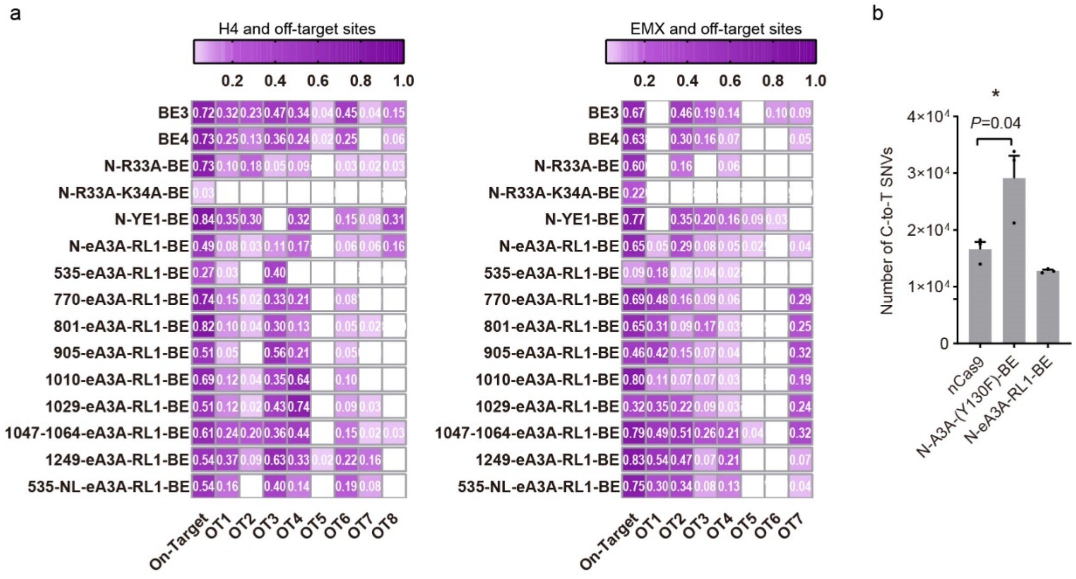

Supplementary Fig. 15 The gRNA-dependent DNA off-target effects and WGS analysis of representative eA3A-RL1-derived CBEs. a, Heatmaps showing gRNA-dependent DNA off-target effects at two endogenous sites H4 and EMX for BE3-no UGI, N-K33A-R34A-BE, N-YE1-BE and representative eA3A-RL1-derived CBEs in purple gradient color. n=3 biologically independent experiments. **b,** Total number of C-to-T (and G-to-A at complementary strand) SNVs relative to the parent sample detected by WGS. The A3A-dervied CBEs N-A3A-(Y130F)-BE were included as positive control. n=3 biologically independent experiments, *P<0.05 with two-tailed unpaired t-test. All values are presented as mean ± s.e.m.

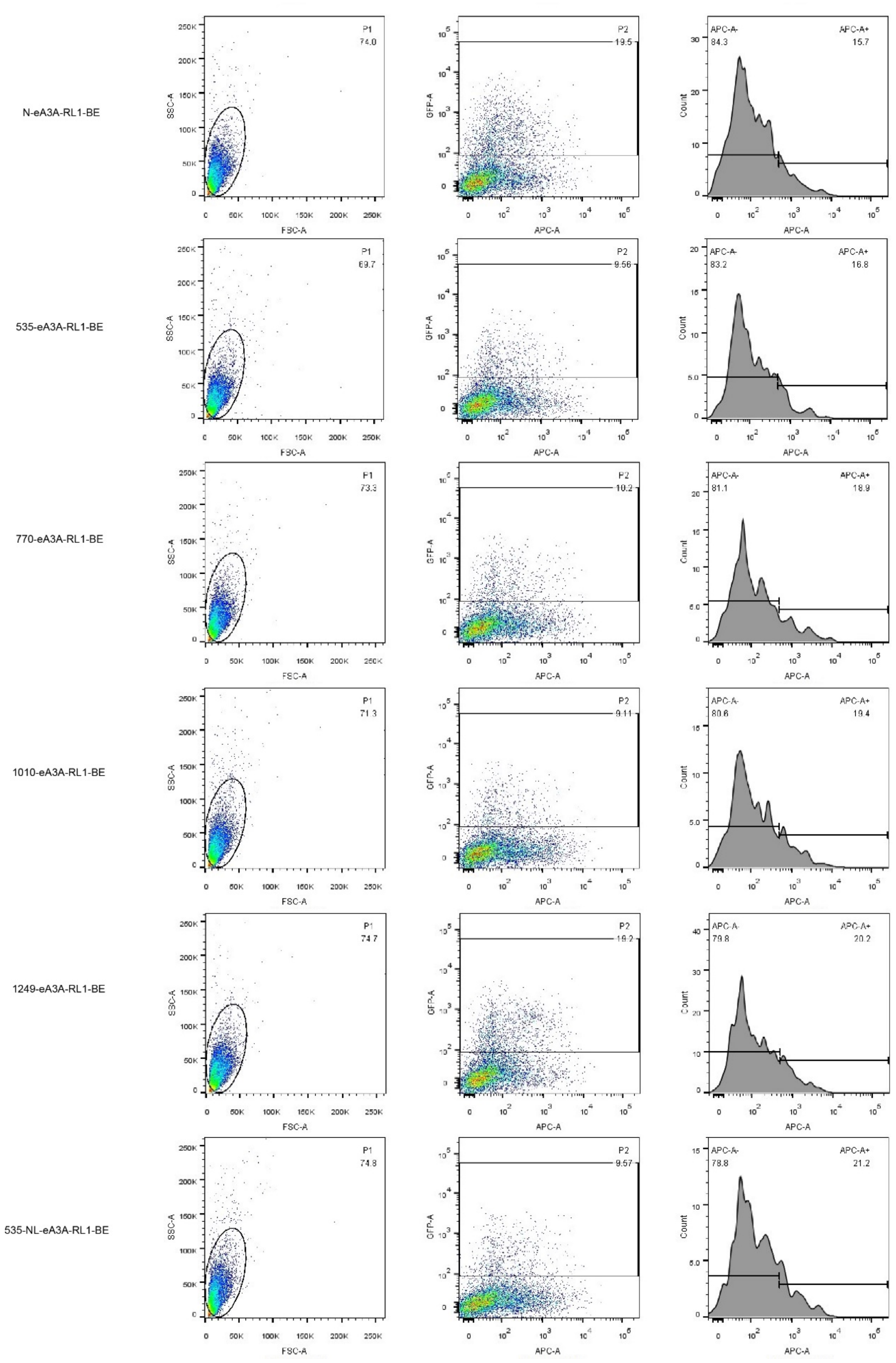

Supplementary Fig. 16 Gating strategy of positive cells and γH2AX antibody fluorescence intensity for flow cytometry analysis. Viable cells were initially selected based

on SSC-A and FSC-A plot. Then cells transfected with enzymes or CBEs were selected based on GFP intensity, while γH2AX antibody fluorescence intensity was analyzed for GFP-positive cells. Representative gating strategy plots for representative eA3A-RL1-derived CBEs were shown. n=4 biologically independent experiments.

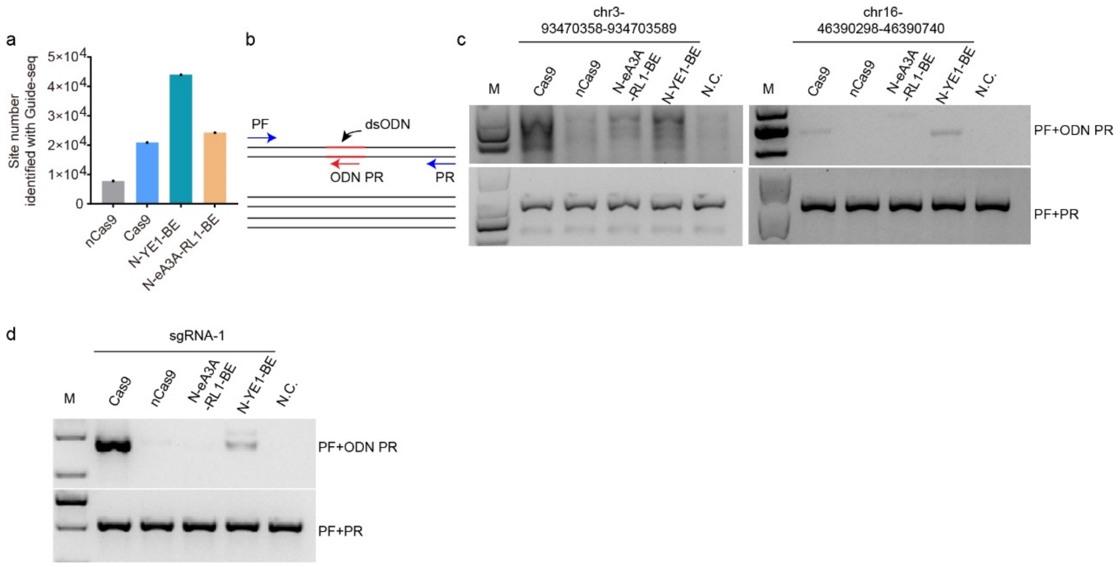

Supplementary Fig. 17 GUIDE-seq analysis for representative CBEs. a, Number of DSBs induced by nCas9, Cas9, N-YE1-BE or N-eA3A-RL1-BE. HEK293T cells were electrotransfected with nCas9, Cas9, N-YE1-BE or N-eA3A-RL1-BE and dsODNs without sgRNA expression. Genomic DNA was collected 72 hours after transfection for GUIDE-seq analysis. **b,** Schemes showing on-target DSB induction activity analysis diagram. Generally, HEK293T cells were electrotransfected with nCas9, Cas9, N-YE1-BE or N-eA3A-RL1-BE and dsODNs and sgRNA targeting specific endogenous site. Then targeted amplification, with one primer located upstream of on-target site and another primer located inside dsODNs for DSB induction analysis. Primer pairs located upstream and downstream of on-target site were used as positive control. The control PCR (PF+PR) and dsODN target PCR (PF + ODN PR) were performed as two separate PCR reactions. **c,** DNA agarose gel analysis of DSB induction activity for Cas9, nCas9, N-eA3A-RL1-BE and N-YE1-BE at two representative regions identified by GUIDE-seq analysis. **d,** DNA agarose gel analysis of on-target DSB induction activity for Cas9, nCas9, N-eA3A-RL1-BE and N-YE1-BE.

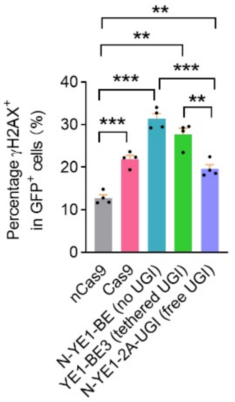

Supplementary Fig. 18 DSB-associated DNA damage analysis for YE1-derived CBEs with distinct UGI structure. Quantification of γH2AX signaling in HEK293T cells transfected with nCas9, Cas9, N-YE1-BE, YE1-BE3 or N-YE1-2A-UGI. N-YE1-BE did not contain UGI domain, YE1-BE3 was in classical CBE structure, with UGI tethered to nCas9, while N-YE1-2A-UGI contained 2A-UGI domain, which could produce free UGI. The percentage of γH2AX positive population within GFP positive cells is shown. n=4 biologically independent experiments, *P<0.05, **P<0.01, ***P<0.001. All values are presented as mean ± s.e.m.

1. Gehrke, J.M., Cervantes, O., Clement, M.K., Wu, Y., Zeng, J., Bauer, D.E., Pinello, L. and Joung, J.K. (2018) An APOBEC3A-Cas9 base editor with minimized bystander and off-target activities. *Nat Biotechnol*, **36**, 977-982.
