# Supplementary material for: Engineering of cytosine base editors with DNA damage minimization and editing scope diversification
Source: Nucleic Acids Res. 2023 Oct 16;51(20):e105. doi: 10.1093/nar/gkad855 (PMC10639057; doi:10.1093/nar/gkad855)
Supplement: gkad855_Supplemental_Files [file gkad855_supplemental_files.zip › Supplementary Table legends-0819.pdf]

Supplementary Table 1: Primer sequences. Primer sequences used for sgRNA synthesis and targeted amplification.

Supplementary Table 2: CBE sequences. Amino acid sequences of CBEs used in this study.

Supplementary Table 3: Representative dsODN integration regions identified by GUIDE-seq analysis.
